# *Ordo ab Chao*: Crystallographic Disorder as a Window into Ionic Liquid Structure

**DOI:** 10.1021/acs.cgd.6c00247

**Published:** 2026-06-02

**Authors:** Joseph Cooper, Marija Scheuren, Lara I. Teodoro, Christopher M. Butch, Raychell A. Jerdo, Kylie M. Allen, Mariana E. Toner, Matthias Zeller, Arsalan Mirjafari, Patrick C. Hillesheim

**Affiliations:** † Department of Chemistry and Physics, 53713 Ave Maria University, Ave Maria, Florida 34142, United States; ‡ Department of Chemistry, 14828 State University of New York at Oswego, Oswego, New York 13126, United States; § Department of Chemistry, 8522 Purdue University, West Lafayette, Indiana 47907, United States; ∥ Department of Chemistry, 6049 Illinois State University, Normal, Illinois 61761, United States

## Abstract

Despite being intended to resist crystallization, ionic liquids (ILs) quite frequently form solids, albeit with low melting points. The design motifs that lower melting points can leave measurable signatures in the solid state, manifesting as crystallographic disorder, and often give rise to metastable liquid states. While sometimes treated as experimental complications, such features provide a valuable window into the structural origins of IL phase behavior. Herein, we report a crystallographic study of a series of benzylated ILs that crystallize either directly or from long-lived supercooled melts. Single-crystal X-ray diffraction, supported by computational modeling and statistical analyses, provides structural evidence that conformational heterogeneity and interaction degeneracy can impede the propagation of long-range order. Competition among multiple energetically similar CH_3_···π and related π-mediated interactions generates complex crystallographic landscapes in which multiple local arrangements coexist, leading to the formation of metastable liquid states prior to crystallization. Notably, across the series of structures examined, cations that successfully crystallize converge on a recurring effective molecular volume, achieved through static packing, conformational flexibility, or crystallographic disorder. Viewed in this context, disorder is not incidental but mechanistically informative, encoding how ILs accommodate volume, distribute interactions, and navigate the boundary between liquid persistence and crystalline order.

## Graphical abstract

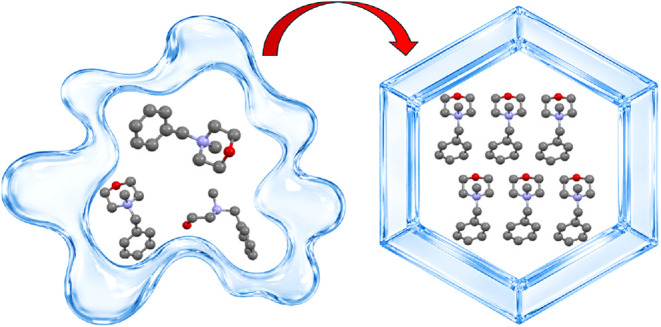

## 1 Introduction

Task-specific ionic liquids (TSILs) constitute a subset of ILs with specific functional groups tethered to the cation or anion.^1^ Having the well-established benefits of conventional ILs,^2,3^ TSILs have seen a prolific increase in reports spanning numerous fields of study including separations,^4^ catalysis,^5^ and biomass processing.^6^ Several fundamental reviews on the topic of task-specific ILs have been written and are a suggested starting point for those so inclined.^7,8^ Among the diverse functional groups employed in TSIL design, benzyl moieties represent a particularly intriguing case as the aromatic ring introduces π-based interactions that fundamentally alter both liquid-state organization and solid-state packing behavior.

Benzyl-functionalized ILs exemplify a central design paradox in IL chemistry: incorporation of π containing moieties within molecules can introduce new supramolecular synthons.^9^ Logically, one might conclude that this would result in the formation of additional noncovalent interactions (NCIs), such as cation-π or π–π stacking interactions,^10^ and thus increase melting points or viscosity which may be considered unfavorable. Indeed, the prototypical benzyl-containing IL, namely 1-benzyl-3-methylimidazolium bis­(trifluoromethanesulfonyl)­imide, or [BzMim]­[NTf_2_], has been rigorously examined with respect to the impact of the benzyl ring on thermal properties, demonstrating how π interactions affect thermal behavior when contrasted with dialkylated ILs.^11^ However, these π interactions allow for expanded applications of these ILs, such as for separations.^12^

The persistence of the liquid state in [BzMim]­[NTf_2_] is perhaps intriguing given the presence of additional π-based interactions, which might otherwise be expected to promote efficient molecular packing and crystallization. The benzyl ring’s influence on phase behavior, however, proves to be anion-dependent and structurally nuanced. While the archetypal IL 1-butyl-3-methylimidazolium or [Bmim]­[PF_6_] is a room-temperature liquid, its benzylated analogue, [BzMim]­[PF_6_], crystallizes, adopting an extended solid-state structure that exhibits multiple π-based interactions.^13^ This anion-dependent crystallization behavior suggests that the energetic benefit of π-mediated packing is balanced against geometric frustration imposed by the benzyl substituent, a balance that shifts depending on anion size, geometry, and charge distribution.^14^

The tendency toward liquid persistence in benzylated ILs manifests most dramatically in their pronounced supercooling behavior.^15,16^ Recent spectroscopic and theoretical studies suggest that low torsional barriers between the benzyl and imidazolium rings, coupled with frustrated π-stacking interactions, give rise to locally organized domains unable to propagate into long-range crystalline order.^17^ Such structural frustration provides a structural rationale for the exceptional glass-forming ability and persistent supercooling observed in benzylated ILs. This raises a fundamental question: how do π-based interactions, conformational flexibility, and molecular volume collectively govern the competition between supercooling and crystallization in benzylated ILs? Answering this requires direct structural characterization of both stable and metastable crystalline states to provide clarity with respect to the design of ILs broadly.

Despite the increasing routineness and accessibility of single-crystal X-ray diffraction (SCXRD), its application within the field of ILs remains comparatively underdeveloped.^18^ It is worth noting, however, that the seminal work defining modern ILs relied on single-crystal structures, establishing an early structural foundation for the field.^19^ As crystallographic methods have continued to advance, a growing number of studies have leveraged diffraction techniques to extract detailed insight into the structural origins of IL physicochemical behavior.

Notably, Zürner et al. combined SCXRD with differential scanning calorimetry (DSC) to examine phase behavior and solid-state structures in Bmim-based ILs.^20^ By crystallizing these materials directly from supercooled melts, they accessed metastable crystalline phases, providing structural snapshots of states immediately preceding the liquid-to-solid transition. Critically, this revealed metastable crystalline phases and allowed for correlation to thermal events with symmetry changes, conformational disorder, and solid-state organization. Complementary work by Paulechka et al. examined the structures of both the butyl and ethylimidazolium-based [NTf_2_]^−^ ILs.^21^ Their study explored several key structural themes, including polymorphism, the cis–trans conformational behavior of the [NTf_2_]^−^ anion, and explicit links between liquid-state organization and the resulting crystalline phases. Together, these studies established that the solid-state structures of ILs, particularly those obtained from metastable conditions, encode information about the conformational and interaction landscapes that govern liquid-state behavior.

One recurring feature emerging from the structural study of ILs is the frequent presence of crystallographic disorder.^18^ This behavior is not unexpected as ILs are deliberately designed to frustrate efficient crystallization through the introduction of energetically low-lying conformational states, which often manifest as positional or conformational disorder in the solid state.^22^ In the study by Zürner et al.,^20^ multiple conformations of the butyl chain in the imidazolium cation were observed with refined occupancies changing systematically as a function of temperature, providing crystallographic evidence for temperature-dependent conformational changes within the lattice. Notably, as the temperature of the crystal structures were varied, the refined occupancies of these conformations changed systematically, consistent with temperature-dependent conformational populations and providing a crystallographic signature of underlying molecular motion within the lattice.

Beyond static disorder models, the atomic displacement parameters (ADPs) obtained from crystallographic refinement provide additional insight into molecular motion within the crystal lattice.^23^ For example, in [Bmim]-based ILs, enlarged displacement ellipsoids associated with terminal methyl groups along the butyl chain are commonly observed, reflecting enhanced local mobility relative to atoms closer to the imidazolium core. Such trends in ADP magnitude and anisotropy offer qualitative indicators of internal molecular flexibility and dynamic behavior. Taken together, analysis of both crystallographic disorder and ADPs allows insight beyond a purely static description of the solid state, instead capturing how ILs accommodate molecular motion and conformational diversity.^24^ Taken together, analysis of both crystallographic disorder and ADPs captures how ILs accommodate molecular motion and conformational diversity even in the solid state.

Fundamental questions remain regarding how aromatic π-interactions shape the energetic landscapes governing crystallization in benzyl-functionalized systems. Our group has employed crystallography as a central tool for IL structural characterization and rationalizing structure–property relationships.^25,26^ Our prior work has demonstrated how aromatic substituents can influence on the physicochemical behavior of ILs.^27–29^ We obtained several single crystals of structurally related, benzyl-functionalized derivatives under diverse conditions, including direct crystallization from long-lived supercooled melts. Collectively, these samples provide an opportunity to examine how π-based interactions, conformational flexibility, and molecular volume collectively govern the competition between liquid persistence and crystalline order. By examining crystallographic disorder, ADPs, and effective molecular volume across aromatic and aliphatic cation frameworks, we demonstrate how subtle differences in interaction topology and conformational flexibility govern packing frustration, supercooling, and phase stability in ILs.

The compounds (Figure 1) probe specific structural variables. Two systems, [2-MetThia]­[NTf_2_] and [Morph]­[NTf_2_], crystallized directly from long-lived supercooled liquids, providing structural snapshots of metastable states immediately preceding crystallization. Serendipitously, [Morph]­[NTf_2_] crystallizes in a commensurately modulated system allowing insight into the movement of molecules prior to crystallization. Analysis of these new systems enabled reinterpretation of a previously reported crystal ([BzMim]­[Br]) exhibiting analogous supercooling behavior,^30^ revealing the role for molecular volume, interaction frustration, and cation conformations in the persistence of supercooled ILs. In parallel, electronic modulation of the cationic framework was explored through nitro substitution, while replacement of the benzyl group with a methylcyclohexyl moiety afforded a useful contrast between π-rich aromatic systems and more conformationally flexible, aliphatic pendant moieties. Collectively, these structures highlight how subtle variations in cation architecture manifest in crystallographic disorder, molecular motion, and phase stability, underscoring the value of crystallography as a lens for understanding the complex behavior of ILs.

**Figure 1:**
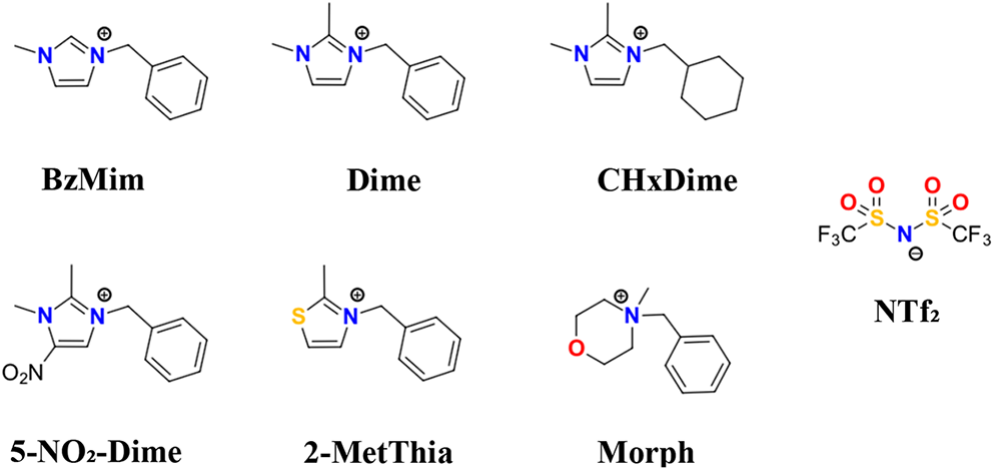
Depiction of the structures of the cations and abbreviations used in the narrative. The [NTf_2_]^−^ anion is also shown for clarity.

## 2 Materials and Methods

### 2.1 Chemicals

All chemicals were purchased in the highest purity available and used as received without further purification. 2-Methylthiazole, benzyl bromide, and (bromomethyl)­cyclohexane were obtained from Ambeed, Inc. *N*-Methylmorpholine was purchased from TCI Chemicals. 1,2-Dimethyl-5-nitroimidazole and lithium bis­(trifluoromethanesulfonyl)­imide ([NTf_2_]^−^) were obtained from Chem-Impex. 1,2-Dimethylimidazole was purchased from Sigma-Aldrich. All solvents were obtained from Fisher Scientific.

### 2.2 Spectroscopy

^1^H, ^13^C and ^19^F NMR analyses were performed on a Bruker 500 MHz NMR at 295 K with the chemical shifts (δ) notated as parts per million (ppm) and referenced to the residual ^1^H signal of DMSO-*d*_6_ as a solvent at room temperature. The following abbreviations were used to explain NMR peak multiplicities: s = singlet, d = doublet, t = triplet, q = quarter, m = multiplet.

### 2.3 Synthesis and Characterization

#### 2.3.1 General Procedure for Synthesis of ILs

The heterocyclic precursor (1.0 equiv) was dissolved in THF (minimal volume), and benzyl bromide (1.2 equiv) was added. The reaction mixture was stirred at 50 °C for 16 h. The resulting halide salt was collected by filtration, washed with ethyl acetate, and dried under vacuum.

The halide salt was dissolved in water (minimal volume) for anion metathesis, and lithium bis­(trifluoromethanesulfonyl)­imide (1.2 equiv) was added. After stirring for 2 h, the product was extracted with ethyl acetate. The organic layer was washed with deionized water until a negative halide test (AgNO_3_) was obtained, dried over anhydrous Na_2_SO_4_, and concentrated under reduced pressure to afford the IL product.

##### 2.3.1.1 1-Benzyl-1,2-dimethylimidazolium Bis­(trifluoromethanesulfonyl)­imide ([Dime]­[NTf_2_])

Single crystals suitable for X-ray diffraction were obtained by slow evaporation of a THF solution. ^1^H NMR (500 MHz, DMSO-*d*_6_) δ 7.7 (d, *J* = 2.0 Hz, 1H), 7.7 (d, *J* = 2.1 Hz, 1H), 7.4 (m, 5H), 5.4 (s, 2H), 3.8 (s, 3H), 2.6 (s, 3H); ^13^C NMR (125 MHz, DMSO-*d*_6_) δ 145.1, 135.1, 129.4, 128.2, 123.2, 121.7, 121.2, 118.7, 51.1, 35.3, 9.9; ^19^F NMR (282 MHz, DMSO-*d*_6_) δ −78.6.

##### 2.3.1.2 1-Benzyl-1,2-dimethyl-5-nitroimidazolium Bis­(trifluoromethanesulfonyl)­imide ([5-NO_2_-Dime]­[NTf_2_])

Single crystals suitable for X-ray diffraction were obtained by slow evaporation from acetone. ^1^H NMR (500 MHz, DMSO-*d*_6_) δ 9.2 (s, 1H), 7.4 (m, 5H), 5.6 (s, 2H), 4.0 (s, 3H), 2.8 (s, 3H); ^13^C NMR (125 MHz, DMSO-*d*_6_) δ 148.9, 133.9, 129.5, 129.3, 128.4, 125.0, 121.2, 118.7, 52.3, 35.7, 11.1; ^19^F NMR (282 MHz, DMSO-*d*_6_) δ −79.4.

##### 2.3.1.3 *N*-Benzyl-*N*-methylmorpholinium Bis­(trifluoromethanesulfonyl)­imide ([Morph]­[NTf_2_])

Single crystals suitable for X-ray diffraction formed from the neat supercooled liquid after standing ambient temperature for two years.^1^H NMR (500 MHz, DMSO-*d*_6_) δ 7.6 (m, 5H), 4.7 (s, 2H), 4.0 (m, 4H), 3.5 (ddd, *J* = 13.3, 9.0, 4.3 Hz, 2H), 3.3 (d, *J* = 12.7 Hz, 2H), 3.1 (s, 3H); ^13^C NMR (125 MHz, DMSO-*d*_6_) δ 133.6, 130.8, 129.4, 127.5, 123.8, 121.2, 118.7, 116.1, 68.3, 60.3, 59.1, 45.5; ^19^F NMR (282 MHz, DMSO-*d*_6_) δ −79.5.

##### 2.3.1.4 1-Benzyl-2-methylthiazolium Bis­(trifluoromethanesulfonyl)­imide [2-MetThia]­[NTf_2_]

Single crystals suitable for X-ray diffraction formed from the neat supercooled liquid after standing at ambient temperature for several weeks. ^1^H NMR (500 MHz, DMSO-*d*_6_) δ 8.4 (d, *J* = 4.0 Hz, 1H), 8.2 (d, *J* = 4.0 Hz, 1H), 7.4 (m, 5H), 5.7 (s, 2H), 3.0 (s, 3H); ^13^C NMR (125 MHz, DMSO-*d*_6_) δ 172.4, 137.7, 133.6, 129.6, 129.4, 128.6, 123.7, 121.3, 118.7, 55.2, 15.9; ^19^F NMR (282 MHz, DMSO-*d*_6_) δ −78.7.

##### 2.3.1.5 1-(Cyclohexylmethyl)-1,2-dimethylimidazolium Bis­(trifluoromethanesulfonyl)­imide [CHxDime]­[NTf_2_]

Single crystals suitable for X-ray diffraction were grown by vapor diffusion of Et_2_O into a saturated CHCl_3_ solution of the compound. Samples were stored at −20 °C for several days before suitable crystals formed. ^1^H NMR (500 MHz, DMSO-*d*_6_) δ 7.6 (m, 2H), 4.0 (d, *J* = 7.5 Hz, 2H), 3.8 (s, 3H), 2.6 (s, 3H), 1.7 (m, 6H), 1.1 (m, 5H); ^13^C NMR (125 MHz, DMSO-*d*_6_) δ 144.9, 122.7, 121.9, 121.2, 118.7, 53.5, 38.1, 35.1, 29.7, 26.1, 25.4, 9.7; ^19^F NMR (282 MHz, DMSO-*d*_6_) δ −79.6.

### 2.4 Single Crystal Diffraction

Single crystals of compound were coated with Parabar 10312 or Fomblin oil and transferred to the goniometer of either a Bruker D8 Quest or Quest Eco diffractometer with a PhotonII or PhotonIII charge-integrating pixel array detector (CPAD) and either Mo-Kα sealed X-ray tube or a Cu-Kα radiation microsource X-ray tube. Examination and data collection were performed at 100 to 150 K as specified within the CIF file. Data were collected, reflections were indexed and processed, and the files scaled and corrected for absorption using APEX3 ^31^ and SADABS.^32^

For all compounds, the space groups were assigned and the structures were solved by direct methods using XPREP within the SHELXTL suite of programs^33,34^ and refined by full matrix least-squares against *F* ^2^ with all reflections using Shelxl^35^ using the graphical interfaces Olex2 ^36^ and ShelXle.^37^ H atoms were positioned geometrically and constrained to ride on their parent atoms. C–H bond distances were constrained to 0.95 Å for aromatic C–H moieties, and to 0.99 and 0.98 Å for aliphatic CH_2_ and CH_3_ moieties, respectively. Methyl H atoms were allowed to rotate, but not to tip, to best fit the experimental electron density. *U*_iso_(H) values were set to a multiple of *U*_eq_(C) with 1.5 for CH_3_ and 1.2 for C–H and CH_2_ units, respectively.

For [Morph]­[NTf_2_], the structure is commensurately modulated by tripling of the *a*-axis. Inversion of the major moiety of disordered anions and other slight modulation breaks exact translational symmetry. The structure also emulates space group *Pnma*. Exact mirror symmetry (for the cations) and inversion symmetry (of the anions) is broken by partial ordering of anions and cations (exact 1:1 disorder would be required for *Pnma* symmetry for the anions; exactly identical disorder ratio would be required for exact translational symmetry).

Two of three anions and one or three cations were refined as disordered. The disordered moieties were each restrained to have similar geometries (for the anions) and to have similar geometries as a not disordered equivalent unit (for the cation). *U_ij_* components of ADPs for disordered atoms closer to each other than 2.0 Å were restrained to be similar. Subject to these conditions the occupancy ratios refined to 0.825(3) to 0.175(3) (anion 1), 0.515(15) to 0.485(15) (cation 2) and 0.759(3) to 0.241(3) (anion 3).

For [2-MetThia]­[NTf_2_], the structure was refined as a two-component twin (twin law [001], [01̅0], [100]) with BASF = 0.2234. The cation was refined as a two-part disorder with occupancies of 0.816(3) and 0.184(3).

For [CHxDime]­[NTf_2_] both the anion and cation have multiple disorder. In the cation, the heterocycle and the cyclohexyl ring are independently disordered, with the bridging methylene group refined as not disordered. For the anion, 3-fold disorder was refined. The position and ADP of C13 and C13C were constrained to be identical. All equivalent disordered moieties were restrained to have each similar geometries. *U_ij_* components of ADPs for disordered atoms closer to each other than 2.0 Å were restrained to be similar. Subject to these conditions the occupancy ratio refined to 0.684(3) to 0.316(3) for the heterocycle, to 0.578(4) to 0.422(4) for the cyclohexyl ring, and to 0.630(3) to 0.1275(12) to 0.242(3) for the anion.

Complete crystallographic data, in CIF format, have been deposited with the Cambridge Crystallographic Data Centre. CCDC 2531643–2531647 contains the supplementary crystallographic data for this paper. These data can be obtained free of charge from The Cambridge Crystallographic Data Centre via www.ccdc.cam.ac.uk/data_request/cif.

### 2.5 Crystallographic Measurements and Images

Hirshfeld surfaces, interaction percentage, and fingerprint plots were calculated and produced using *CrystalExplorer25*.^38^ Interaction energies were calculated using *CrystalExplorer*’s parametrized CE-1p-B3LYP level of theory.^39^

Mercury^40^ was used for calculation of the torsion angles and to make the relevant images of structures. PLATON^41^ was used for the study of atomic displacement parameters. Enrichment ratios were calculated following previously reported methods using the interaction values from Hirshfeld surface analysis.^42^

In brief, enrichment ratios (ERs) provide a statistical measure of whether specific intermolecular contacts occur more or less frequently than expected based on the relative surface areas of the interacting atom types. For example, although H···H contacts typically constitute the largest fraction of interactions in organic ionic liquids, enrichment ratios contextualize these values by indicating whether such contacts are statistically favored (*E* > 1) or disfavored (*E* < 1) relative to a random distribution. Values greater than 1 suggest preferential contact formation, whereas values less than 1 indicate interactions that are avoided within crystal packing.

Effective isotropic stiffness constants (*k*_iso_) were calculated from equivalent isotropic displacement parameters (*U*_eq_) assuming a harmonic approximation, using *k*_iso_ = *k*_B_ *T*/*U*_eq_.^43^ Here, *k*_B_ is the Boltzmann constant and *U*_eq_ represents the mean square displacement. The resulting values (in units of N/m) provide site-specific measures of lattice confinement, decoupling thermal motion from the intrinsic potential well width.

The measured torsion angles and the subsequent discussion follows on a modification of previous literature on related topics.^44^ Two torsion angles are discussed as shown in Figure 2. The tabulated torsion angles for all compounds are shown in the Supporting Information.

**Figure 2:**
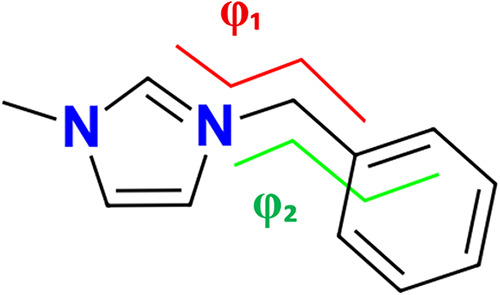
Depiction of the two torsion angles discussed and measured in this study.

## 3 Results and Discussion

The crystals reported herein represent a set of structurally informative snapshots that collectively illuminate how IL design relates to crystallinity. Each structure provides an opportunity to examine a specific feature that may either promote crystallization or, as is often the case, hinder the emergence of long-range order. For clarity within the discussion, static disorder refers to the crystallographic modeling of multiple discrete occupancies within the lattice.^45^ Modulation, by contrast, denotes a periodic structural variation that is not fully described by the basic unit cell.^46^

[Dime]­[NTf_2_] serves as an ordered benzylated reference point, while [5-NO_2_-Dime]­[NTf_2_] probes the effect of electronic modulation on intermolecular interactions. [2-MetThia]­[NTf_2_] and [Morph]­[NTf_2_] crystallized from long-lived supercooled liquids and therefore provide structural snapshots of metastable states immediately preceding crystallization, with the latter additionally exhibiting commensurate modulation. The previously reported [Mim]­[Br] is revisited as a related supercooled system for comparison, whereas [CHxDime]­[NTf_2_] provides a nonaromatic, isovolumetric contrast that helps separate steric and volumetric effects from π-mediated packing. Taken together, these structures allow us to compare how crystallization pathway, disorder type, conformational flexibility, and interaction topology contribute to frustrated packing in benzylated ionic liquids.

### 3.1 Cation–π Interactions: Anion and Electronic Changes

The [Dime] cation, one of the iconic IL motifs and, relevant to the discussions herein, represents the “ordered ideal”: a structure without any disorder making it our reference point. Somewhat surprisingly, despite the longstanding interest in benzyl-substituted ILs, [Dime] itself has only been reported twice previously, with one of these originating from the “halcyon” era of ILs in the late 1990s.^47,48^ Additionally, there also exist two reported crystals containing the [Dime] cation, allowing us to draw richer structural insight.^49,50^ To remain within the general context of classical ILs, we focus here on the crystal of the iodide salt, [Dime]­[I], allowing for contrast of anions. In addition of anion influence, the electronic modulation on intermolecular interactions is further examined through comparison with the nitro-substituted derivative ([5-NO_2_-Dime]). Together, these three structures provide a compact but informative platform for understanding how substitution and anion identity shape packing preferences in benzylated ionic liquids (Figure 3).

**Figure 3:**
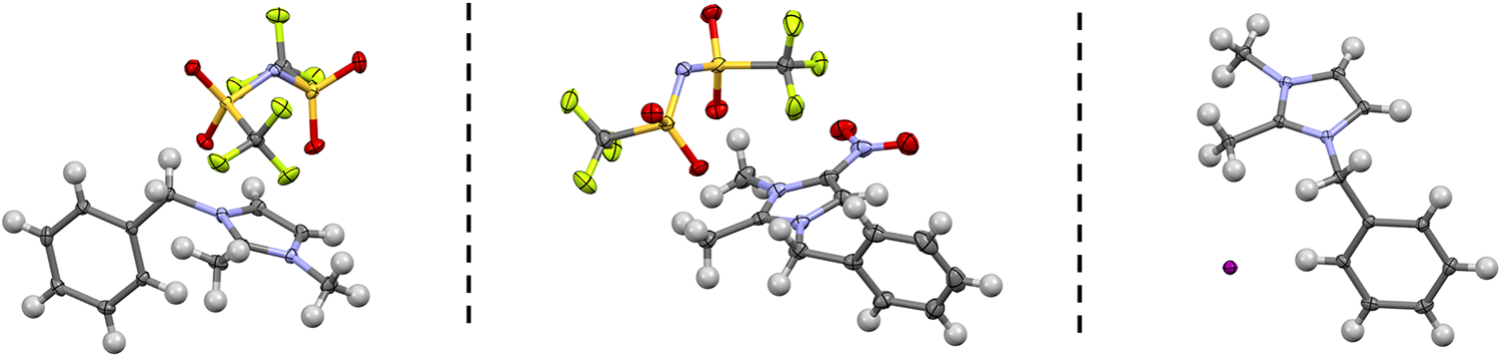
Asymmetric units of [Dime]­[NTf_2_], [5-NO_2_-Dime]­[NTf_2_], and the previously reported [Dime]­[I].^50^ Thermal ellipsoids shown at the 50% probability level.

The enrichment ratio (ER) analysis highlights a pronounced redistribution of preferred contacts upon introduction of the nitro substituent (Table 1). In the unsubstituted [Dime] structure, moderate enrichment of H···C (π-type) contacts (*E*_HC_ = 1.44) indicates a statistically meaningful contribution from cation–cation and cation−π interactions involving the benzyl ring. In contrast, these interactions are strongly suppressed in [5-NO_2_-[Dime]] (*E*_HC_ = 0.45), coincident with a marked shift toward cation–anion contacts. This redistribution reflects the electron-withdrawing nature of the nitro group, which depletes electron density from the aromatic system and increases the electrostatic attraction between the cation and the [NTf_2_]^−^ anion.

**Table 1:** Enrichment Ratios (*E*_XY_) for Key Intermolecular Contacts across All Crystallographically Independent Cation Environments Discussed in the Manuscript^a^

Cation	H···H	H···C	C···C	C···N	H···O	H···F	C···O	C···F	N···O	N···F	H···Br	H···I	C···Br	C···I	H···S	C···S
[Dime][NTf_2_]	0.57	1.44	0.00	0.18	1.56	1.69	0.80	0.47	1.79	0.30					1.79	0.00
[5-NO_2_-Dime][NTf_2_]	0.69	0.45	0.00	2.15	1.66	1.28	0.49	3.77	0.70	1.20					1.96	0.00
[2-MetThia][NTf_2_]	0.45	1.74	0.00	0.26	1.68	1.70	0.67	0.19	1.45	0.00					0.90	0.00
[CH_*x*_Dime][NTf_2_]	0.85	0.00	0.00	0.00	1.40	1.38	1.39	5.03	2.04	1.64					0.00	0.00
[Dime][I]*	1.06	0.61	3.19	2.95								1.28		0.00		
[Mim][Br]—Cat A	1.00	0.94	1.37	2.56							1.24		0.24			
[Mim][Br]—Cat B	0.98	0.98	1.31	2.47							1.29		0.05			
[Morph][NTf_2_]—Cat 1	0.62	1.59	0.00	0.00	1.61	1.62	0.13	0.00	0.00	0.00					0.00	0.00
[Morph][NTf_2_]—Cat 2	0.62	1.61	0.00	0.00	1.61	1.62	0.05	0.00	0.00	0.00					0.00	0.00
[Morph][NTf_2_]—Cat 3	0.63	1.56	0.00	0.00	1.60	1.61	0.18	0.05	0.00	0.00					0.00	0.00

a *­[Dime]­[I] was previously reported.^50^

The electronic change manifests most clearly in the emergence of enriched C···F contacts in [5-NO_2_-Dime] (*E*_CF_ = 3.77), which are less favored in the unsubstituted analogue. These contacts are a combination of the anion···π interactions involving the benzyl ring, supplemented by additional interactions between the π faces of the imidazolium ring and the surrounding anions. Notably, both π faces of the imidazolium core participate in anion association. Thus, the nitro group alters the relative weighting of preferred contacts without enforcing a single, dominant packing motif with respect to cation–anion contacts.

[Dime] shares a similar inclination for anion association with the π faces of the imidazolium ring, as reflected in comparable H···O enrichment ratios for the two compounds. However, a key distinguishing feature of the unsubstituted [Dime] structure is the presence of well-defined, directional H···π interactions involving the benzyl substituent. In particular, the central C2–CH_3_ group of the imidazolium ring engages in H···π contacts with a symmetry-related benzyl ring, while the opposite face of that benzyl ring simultaneously interacts with the aromatic C4–H and C5–H protons of a neighboring imidazolium cation. This arrangement generates an extended chain of H···π interactions propagating roughly along the crystallographic *b* axis, forming a recognizable supramolecular synthon that is absent in the nitro-substituted analogue. These π interactions are not present in [5-NO_2_-Dime], most likely due to the drastic conformational changes in the benzyl moiety. These CH_3_···π motifs are a key interaction within these compounds and are also observed in [2-MetThia] (see Section 3.2.2).

Contrasting [Dime]­[I] with [Dime]­[NTf_2_] provides insight into why anion geometry plays a central role in the development of low-melting, low-viscosity ILs. The extended structure of [Dime]­[I] differs markedly from that of [Dime]­[NTf_2_], with the C2–CH_3_···π synthons observed in the [NTf_2_]^−^ salt replaced by directional C–H···I interactions. In [Dime]­[I], each hydrogen of the central C2 methyl group engages an iodide anion in an approximately colinear arrangement, allowing iodide to maximize hydrogen contacts with surrounding cations. Both the halide and [NTf_2_]^−^ anions are capable of forming hydrogen bonds with the cation framework; however, the larger, conformationally flexible [NTf_2_]^−^ anion can satisfy multiple weak interactions simultaneously while occupying a single spatial region. In contrast, halide anions are more strongly driven toward localized, directional interactions, most commonly hydrogen bonds, which limits their ability to engage directly with extended π surfaces. As a result, halide···π interactions are not observed in these specific systems. From an applications perspective, this suggests that while benzylated cations favor π-interactions, the extent to which these interactions contribute to extended structure, and, by extension, macroscopic properties, depends sensitively on anion shape, interaction multiplicity, and electronic structure of the aromatic domains.

Finally, both the imidazolium and benzyl π systems participate in alternating, offset stacking interactions. While benzene–benzene and benzene-imidazolium stacking is observed, no imidazolium–imidazolium stacking occurs within the crystal. Instead, the statistically enriched C···N value (*E*_CN_ = 2.95) arises from offset imidazolium–benzene stacking interactions (centroid–centroid distance = 3.407 Å).

### 3.2 Crystallographic Insights into Supercooling Behavior

A supercooled liquid represents a metastable state in which a material persists in the liquid phase below the temperature at which crystallization would ordinarily occur. In such systems, relatively small perturbations, (e.g., thermal, mechanical, or environmental) may be sufficient to trigger nucleation and subsequent solidification. ILs are particularly prone to supercooling due to their elevated viscosities and frustrated packing landscapes, although the persistence and stability of the supercooled state can vary widely depending on thermal history, impurities, and molecular architecture.

From a structural standpoint, crystals obtained from supercooled melts offer snapshots of molecular organization immediately preceding crystallization, capturing forms of short-range order that may differ substantially from both the equilibrium liquid and fully ordered crystalline states.^51^ In this context, supercooling is not merely a curiosity of ILs but a structurally informative phenomenon of growing interest, particularly in applications where metastability and delayed phase transitions are desirable.^52^

Three benzylated ILs—[Morph], [2-MetThia], and [Mim]­[Br] (Figure 4)—exhibited supercooling prior to crystallization. Although all three systems ultimately form crystalline solids, they display different supercooled lifetimes and crystallographic signatures. Examination of these compounds as a comparative set provides mechanistic insight into how conformational flexibility, interaction directionality, and packing frustration govern the persistence and eventual collapse of the supercooled state, pointing toward structural principles that may enable the deliberate design of supercooled ILs.

**Figure 4:**
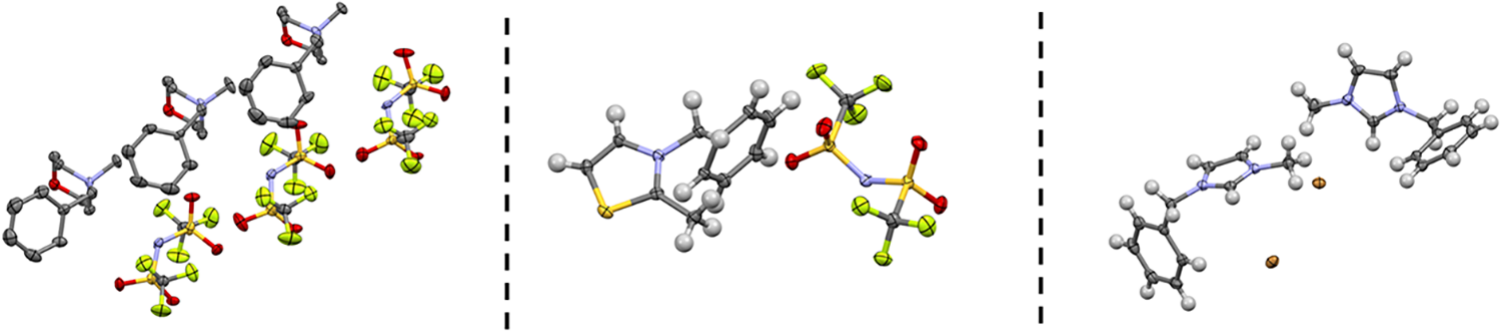
Asymmetric units of [Morph]­[NTf_2_], [2-MetThia]­[NTf_2_], and the previously reported [Mim]­[Br][Bibr ref30] shown with 50% probability ellipsoids. Disorder omitted for clarity. Hydrogens omitted in [Morph] for clarity.

#### 3.2.1 Morpholinium: Conformational Modulation and Long-Lived Supercooling

Shifting from aromatic heterocycles to aliphatic systems such as *N*-benzyl-*N*-methylmorpholinium ([Morph]) brings substantial changes to the physicochemical behavior of ILs.^1^ Aromatic cations benefit from charge delocalization and π-driven interactions, whereas quaternary ammonium ions rely more heavily on steric blocking and local conformational freedom. This shift complicates direct comparison between the species; however, this crystal of [Morph] provides valuable insight into one of the more puzzling, and sometimes frustrating, features of IL: their pronounced tendency to form persistent supercooled liquids. In this regard, [Morph] is unusually illuminating, as the crystalline sample analyzed here formed only after remaining a homogeneous supercooled liquid for nearly two years. Its crystal structure therefore provides a rare snapshot of the conformational ensemble accessible in the metastable liquid immediately prior to crystallization.

The asymmetric unit contains three crystallographically independent cations and three anions (Figure 5). One cation (2) displays a two-site orientational disorder of the benzyl fragment (occupancies ≈ 0.52:0.48), indicating that two conformers coexist with nearly equal probability. Two of the three anions likewise exhibit whole-anion orientational disorder; their major components refine near 0.82 in one case and 0.76 in the other. Only one anion is fully ordered. The importance of conformational flexibility of [NTf_2_]^−^ with respect to the formation of low-melting ILs has been well documented at this point.^53^ Interestingly, all the anions are in the trans conformation.

**Figure 5:**
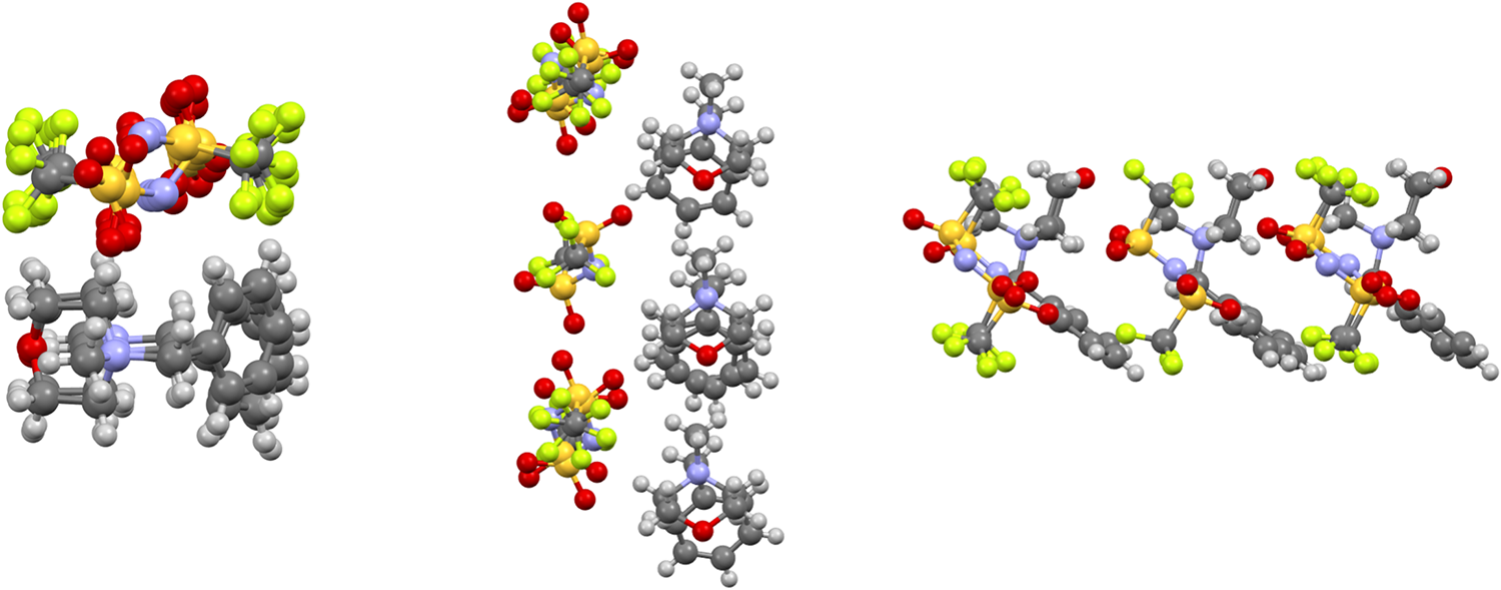
Asymmetric unit of [Morph]­[NTf_2_] viewed down the crystallographic *a*, *b*, and *c* axis. All disordered moieties are shown. The modulation is most readily observed looking down the *a* axis.

Viewing the asymmetric unit along the crystallographic *a* axis (Figure 5) makes the structural origin of the prolonged supercooled behavior apparent: the packing is spatially heterogeneous, arising from a combination of molecular and conformational movement in both the cations and anions, and lacks a single dominant motif capable of efficiently propagating through the lattice. In essence, this crystal structure captures the motions of both ions (see Supporting Information).

The three symmetry-independent cations collectively represent the accessible conformational space of the morpholinium ion. Cations 1 and 3 represent ordered extremes (the top and bottom cations in the modulated stack), whereas cation 2 (the middle cation) captures an intermediate state where the benzyl ring swings between two orientations of almost equal weighting. This benzyl mobility produces a measurable inflation of the molecular volume. Hirshfeld surface volume (*V*_HS_) indicates that while the ordered cations 1 and 3 occupy 249.7 Å^3^, the disordered cation 2 occupies 270.1 Å^3^ (see Table 2). While an ∼8% volume increase may appear numerically small, its impact on packing efficiency is significant. Notably, this 8% expansion arises solely from the benzyl ring motion and does not capture volume changes due to the translational motion of the cation core (i.e., Cation 1 → 2 → 3 → 1).

**Table 2:** Summary of Volumes for the Modulated [Morph] Structure^a^

species	description	*V*_HS_ (Å^3^)	*V*_static_ (Å^3^)	*V*_swept_ (Å^3^)	Δ*V* (%)
[Morph] Cation 1	Ordered conformer (top of modulation)	249.7	186.49		
[Morph] Cation 2	Disordered benzyl (center modulation)	270.1			+8.2% vs A/C (*V*_HS_)
[Morph] Cation 3	Ordered conformer (bottom of modulation)	249.7			
[Morph] Cation (1→2→3 trajectory)	Full modulation pathway		186.49	220.54	+18.3%
[NTf_2_]^−^ Anion (trajectory)	Translationally modulated		147.41	207.60	+40.8%

a *V*_HS_ is the volume calculated from the Hirshfeld surface; *V*_static_ is a calculated volume based on atomic radii; *V*_swept_ is the volume of the combined interpolated cations in the modulation.

To quantify the total steric requirement of the modulation, including the translational “wobble” of the cation core, we performed a geometric trajectory analysis (see Supporting Information). A baseline static volume was first calculated using the atomic coordinates of Cation 1 and standard Bondi^54^ van der Waals radii (*V*_static_ = 186.49 Å^3^). We then calculated the “swept volume” of the modulation trajectory by interpolating between the overlaid frames of cations 1, 2, and 3. This dynamic swept volume (*V*_swept_) was calculated to be 220.54 Å^3^, an expansion of 18.3% over the static volume, a notable increase in the effective volume of the cation within this crystal. The same analysis was performed for the anions, giving *V*_static_ = 147.41 Å^3^ and *V*_swept_ = 207.60 Å^3^, representing a substantially larger 40.8% increase in effective steric volume (Figure 6). Importantly, these values represent changes in the effective molecular volume arising from modulation, not changes in crystallographic unit cell volume.

**Figure 6:**
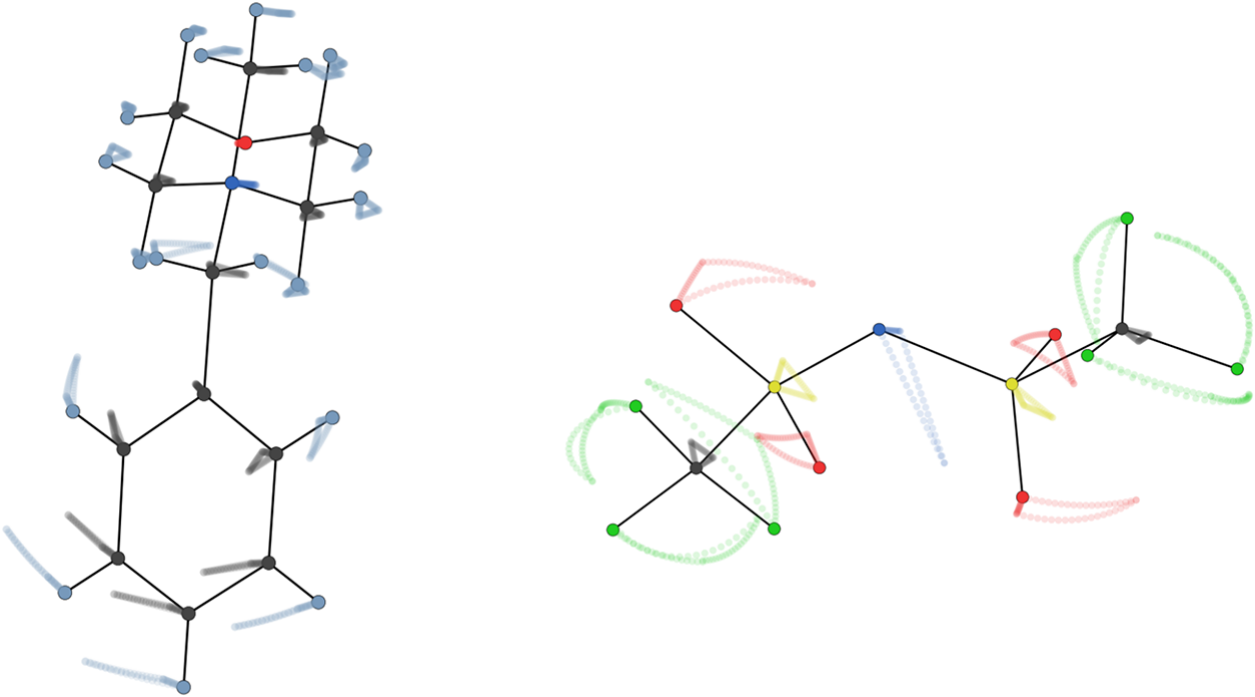
Image showing the movement of the atoms during the interpolated modulation of the [Morph] cation (left) and [NTf_2_] anion (right). Ghost atoms are shown for the intermediate frames for clarity.

These results indicate that modulation dramatically increases the steric footprint and orientational sampling of both ions. Consequently, the local environment must transiently accommodate substantially larger effective ion volumes and while accommodating changing intermolecular contact geometries before periodic order can propagate. This additional configurational and packing frustration likely raises the kinetic barrier to nucleation and growth, providing an explanation for the unusually prolonged supercooled state observed for [Morph].

Notably, despite the substantial motion of the benzyl group and cations themselves, the overall intermolecular interactions among the three cations remain remarkably similar. As shown in Table 1, the interaction enrichment ratios vary only minimally across the independent cations. The H···H enrichment ratios, which reflect cation–cation contacts, are essentially identical at approximately 0.62 for all three. Likewise, cation–anion contacts are largely unchanged, with H···N, H···O, and H···F enrichment ratios remaining consistent at roughly 1.62, 1.61, and 1.62, respectively. The only meaningful deviation occurs for H···C contacts: cation 2 exhibits a slightly higher enrichment ratio of 1.61 compared with 1.59 and 1.56 for cations 1 and 3. These H···C interactions correspond primarily to H···π contacts with the benzyl ring, and the ratio for cation 2 is fully consistent with the observed motion of the benzyl moiety.

Taken together, these observations help rationalize why [Morph] persisted as a supercooled liquid for such an extended period. The presence of nearly isoenergetic cation interaction environments and locally reduced packing efficiencies weaken the cohesive energy of the lattice. Moreover, the extensive disorder of the cations and anions implies that the solid state possesses high entropy, likely indicating a diminished change in entropy of fusion (Δ*S*_fus_) compared to a transition from a more ordered solid. This reduced thermodynamic distinction between the liquid and the solid suggests the crystal is merely a frozen snapshot of the liquid’s conformational ensemble. In such a scenario, characterized by multiple accessible local arrangements and a reduced thermodynamic driving force for ordering, crystallization may be delayed, allowing the IL to persist in a metastable state longer than more rigid systems.

#### 3.2.2 [2-MetThia]: Low-Penalty Cation Disorder and π Interactions

Similar to [Morph], [2-MetThia] persisted as a supercooled liquid prior to crystallization, and several structural parallels between the two compounds help explain this behavior. The two disordered components of [2-MetThia] are shown in Figure 7. Although [2-MetThia] does not display the pronounced modulation evident in [Morph], the crystallographic disorder and supramolecular interactions still provide detail for the observed metastability.

**Figure 7:**
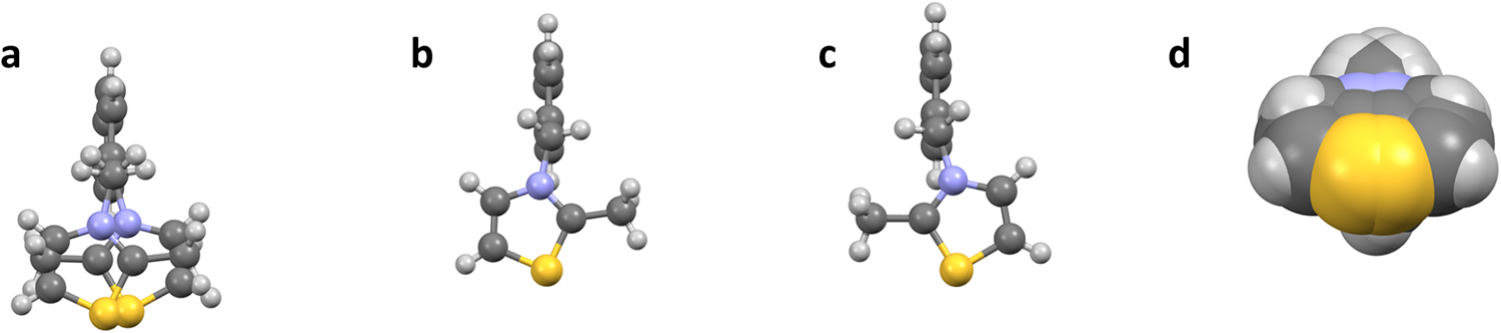
(a) The disordered model of the cation in [2-MetThia]­[NTf_2_]; (b, c) the two components of the disordered model, showing a ring inversion of the thiazolium heterocycle; (d) a space filling model of the disordered model.

Broadly, the disorder of the cation does not dramatically alter the accessible surface of the molecule, nor does the ring-flip introduce substantial geometric distortions such as ring inversions or backbone rearrangements. A space-filling representation of the combined disorder states illustrates that the two orientations are quite similar, with the thiazolium sulfur and π faces of the ring being accessible in both orientations. Consequently, the rotation of the thiazolium–benzyl fragment produces only subtle changes in the local environment, limiting the structural disruption of the disorder. However, these subtle geometric changes manifest in more pronounced energetic changes with respect to interactions (see Section 3.3).

Applying a similar methodology used for [Morph], we examined the changes in accessible volume arising due to the disorder. The Hirshfeld surface volume for the isolated major component was found to be 241.5 Å^3^ and 251.7 Å^3^ for the disordered model, an expansion of +4.2%. Thus, the steric penalty in [2-MetThia] (∼4%) is drastically smaller than that observed in [Morph] (∼18%). This aligns well with the kinetic stability of the supercooled liquids: [Morph] resisted crystallization for years, whereas [2-MetThia] crystallized after only several weeks. While the Hirshfeld volumes of the two cations are comparable for their individual cations, their dynamic footprints differ fundamentally: [2-MetThia] is stabilized by directional π-interactions, whereas [Morph] is frustrated due to increased rotational flexibility and by a larger number of nondirectional, energetically degenerate H···H contacts.

This structural stability is reflected in the enrichment ratios. [2-MetThia] shows strongly enriched H···C contacts (*E*_HC_ = 1.91), consistent with dominant intermolecular C—H···π interactions involving the thiazolium ring protons, the C2 methyl group, and the benzyl π-system. These interactions mirror the supramolecular synthons observed in [Dime], indicating that CH_3_···π and C4/C5—H···π contacts play nontrivial role in forming long-range order (Figure 8). Their persistence across both orientations of the disordered [2-MetThia] cation explains why the disorder is crystallographically tolerated: both conformers preserve the essential H···π motif. The enrichment analysis reinforces the interpretation that these directional interactions provide a structural anchor within the isotropic Coulombic environment, rationalizing the eventual crystallization despite the supercooled history.

**Figure 8:**
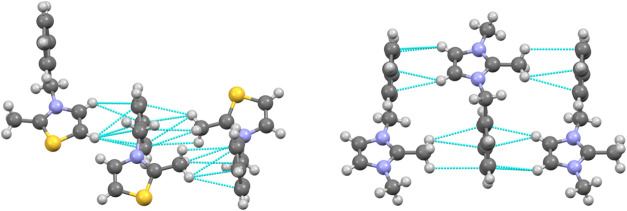
Depiction of the π interactions in [2-MetThia] (left) and [Dime] (right). For [2-MetThia] only the cation from the major part of the disorder is shown, however the ring inversion does not disrupt the formation of these interactions. H···C contacts are shown in light blue dotted lines to a distance of vdW radii +0.3 Å.

Interestingly, the [2-MetThia] crystal also contains a small, twinned component (∼18%), and correcting for this did not remove the cation disorder. Instead, both phenomena coexist, indicating that the lattice accommodates multiple orientation variants at very low energetic cost. This behavior is consistent with a weakly differentiated packing landscape in which several nearly equivalent arrangements compete during nucleation, a hallmark of ILs that resist crystallization and remain supercooled for long periods.

#### 3.2.3 [Mim]­[Br]: Hydrogen Bonding and Competing π-Preferences

The structure of [Mim]­[Br] was previously reported by Peppel et al.,^30^ with the authors noting that the compound persists as a supercooled liquid that crystallizes only after several weeks, mirroring the behavior we observed for [2-MetThia]. With the insights gained from [2-MetThia] and [Morph], and aided by Hirshfeld surface analysis with the corresponding enrichment ratios, we can now propose a structural rationale for the supercooled state of [Mim]­[Br]. The asymmetric unit contains two independent cations (A and B) and two bromide anions, each in subtly different local environments.

First, hydrogen bonding governs anion placement. The H···Br contacts are strongly enriched for both cations, with *E*_HBr_ = 1.24 for cation A and 1.29 for cation B. In contrast, other Br-containing contacts are markedly depleted: *E*_CBr_ is only 0.24 (A) and 0.05 (B), and *E*_NBr_ is 0.32 (A) and 0.00 (B). The remaining interactions—*E*_HH_ ≈ 1.00, *E*_HC_ ≈ 1.00, and *E*_HN_ ≈ 0.8—are essentially statistical or slightly disfavored. Collectively, these values demonstrate that [Br]^−^ overwhelmingly targets hydrogen sites on the cation rather than engaging in Br···π or Br···heteroatom interactions. This describes the now well understood H-bonding behavior in ILs.^55^

Surprisingly, however, the bromide does not form a classical, linear hydrogen bond with the acidic C2–H site. Instead, the [Br]^−^ sits at a C2–H···Br distance of 2.867(3) Å with an angle of 133.37°, deviating from the more linear geometry observed in related systems such as [Bmim]­[Br][Bibr ref56] where the C2–H···Br angle is 163.4°. Rather than acting as a single, directional hydrogen-bond acceptor, the bromide in [Mim]­[Br] occupies a pocket defined by multiple hydrogen donors, including aromatic benzyl hydrogens and the C4/C5–H sites of the imidazolium ring. This multicontact, off-angle coordination provides electrostatic stabilization but lacks the directionality typically associated with the robust hydrogen-bonding synthons.

Second, despite the dominance of hydrogen bonding, the cations still exhibit a clear tendency toward π-type interactions. The *E*_CC_ ratios are enriched for both independent cations (1.37 for A and 1.31 for B), and this effect is even more pronounced for *E*_CN_ (2.56 for A and 2.47 for B). These contacts arise from the imidazolium ring and benzyl fragments approaching symmetry-related aromatic groups and neighboring imidazolium rings. Because the bromide anion is engaged primarily in predominantly nondirectional hydrogen bonding, both π-faces of the cation remain accessible, prompting the system to recover stabilization through C···C and C···N contacts. However, unlike [Dime] and [2-MetThia], [Mim]­[Br] lacks suitably oriented methyl groups (i.e., C2 methyl) capable of forming the aforementioned CH_3_···π interactions. As a result, the π-network is composed of weaker, canted H···π contacts rather than the directional synthons observed in the C2 methylated systems.

Finally, the lack of an energetically dominant synthon, as evidenced by the combination of off-angle hydrogen bonding and weaker secondary π-interactions, results in two crystallographically distinct cations in the asymmetric unit. These cations differ primarily in the torsion angles of the benzyl moiety, resulting in subtly different interaction environments. Cation B, for example, exhibits slightly more enriched H···Br contacts and more depleted C···Br contacts than cation A, reflecting small variations in how the bromide engages the surrounding hydrogen framework. Although these differences are modest in magnitude, they indicate that no single packing motif is sufficiently favorable to propagate uniformly throughout the lattice. Instead, the crystal accommodates multiple nearly isoenergetic local arrangements, producing a frustrated packing landscape analogous to that observed in [Morph] and [2-MetThia].

Consistent with this interpretation, overlaying the two independent cations reveals a modest but measurable geometric distinction arising from benzyl rotation, corresponding to a volumetric expansion of approximately 5.3% (Figure 9). This value closely matches the steric penalty associated with the ring-flip disorder in [2-MetThia] and, while significantly smaller than the extensive modulation observed in [Morph], nonetheless indicates that the [Mim]­[Br] lattice can accommodate multiple cation geometries of comparable energy.

**Figure 9:**
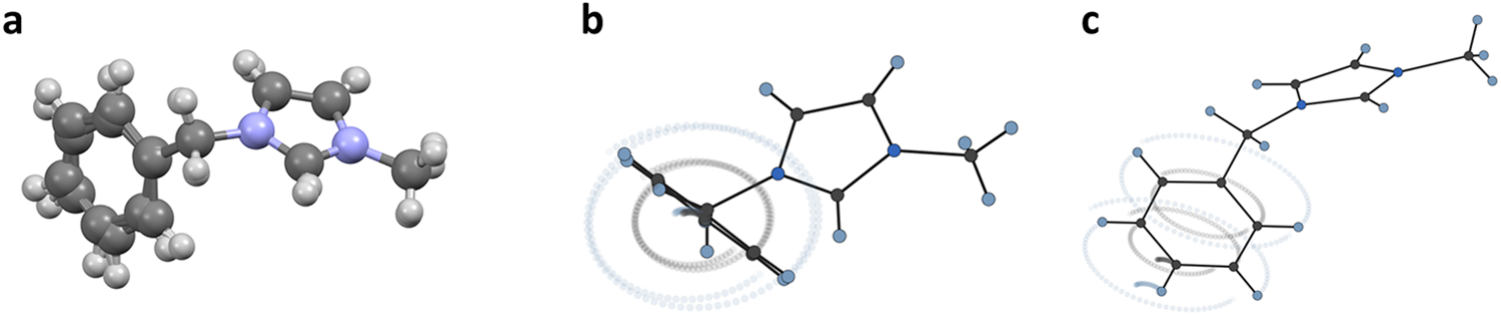
(a) Depiction of the overlaid cations in [Mim]­[Br]; (b, c) representation of the interpolated structures for the rotation of the benzene moiety. Ghost atoms are shown for the intermediate frames for clarity.

To quantify the steric impact of this flexibility, we generated an interpolated set of frames for the rotation of the benzyl moiety in [Mim]­[Br] and calculated the sweep volume of this rotation (Figure 9). The static volume of the cation was calculated to be 164.2 Å^3^ with a sweep volume of 228.1 Å^3^, an increase of ∼39%. This result reveals important structural clues about this class of ILs. The benzyl moiety, through rotation about the C_ipso_–C_benzyl_ bond, results in a notable increase in effective steric volume. Moreover, we note that this simple approximation of volume does not account for unrestricted rotation of the entire benzyl moiety, which would further increase the cation volume. Nevertheless, this 39% steric expansion provides a structural rationale for the stability of the supercooled liquid state. This suggests that for the supercooled liquid to crystallize it must overcome a significant entropic barrier associated with the “locking” of this large dynamic volume.

Taken together, the enrichment ratio data, hydrogen-bond geometry, and volumetric expansion support a clear interpretation: hydrogen bonding in [Mim]­[Br] provides strong but spatially diffuse stabilization, while the extended π-network lacks a robust, directional synthon capable of reinforcing long-range order. The lattice therefore stabilizes multiple local arrangements of similar energy, impeding the rapid selection of a unique packing motif and enabling [Mim]­[Br] to persist as a supercooled liquid for extended periods prior to crystallization.

#### 3.2.4 Comparative Analysis of Supercooling Mechanisms

As alluded to earlier, the change from aliphatic to aromatic cation brings about important structural changes. We can use these to further rationalize the behavior of these supercooling species. From the perspective of sterics, in [2-MetThia], the flat, aromatic thiazolium ring exposes the cationic nitrogen center to the anion via the π-face. This accessibility allows the [NTf_2_]^−^ anion to dock efficiently above and below the heterocycle, facilitating close approach of the anionic oxygens to the cationic center and stabilizing the lattice through favorable anion-π and Coulombic interactions. This is evidenced by the high *E*_NC_ of 4.66, along with the increased *E*_FC_ ratio of 1.83. Ultimately, these strong Coulombic interactions help lead to the formation of a crystalline state.

For [Mim]­[Br], the smaller halide anions do not interact with the π system of the cations, preferring the more energetically favorable H···Br interactions with the cation aromatic hydrogens. With the open π faces of the cation, the [Mim] cation attempts to form π interactions, but given the lack of readily available methyl groups, as observed in [2-MetThia] and [Dime], is not capable of forming the observed H···π interactions, frustrating lattice formation. Thus, [Mim]­[Br] provides a useful point of contrast by evaluating the impact of anion structure. Namely, the larger and more complex [NTf_2_]^−^ anion can form multiple interactions with a single cation while bromides will tend to form the more energetically preferably hydrogen bonds.

These observations provide a crucial distinction from the [Morph] system. While [Morph] relies on a fuzzy, spherical electrostatic field, both [2-MetThia] and [Mim]­[Br] possess structural anchor points in the sulfur atom for [2-MetThia] and the acidic central C2—H for [Mim]­[Br]. These specific anchoring points likely reduces the entropic barrier to nucleation; once the sulfur or C2—H finds the anion in the melt, the rest of the lattice snaps into place via either the π-stacking channels or through H-bonding, explaining the shorter supercooling lifetime (weeks vs years). Moreover, both [2-MetThia] and [Mim] share similar observable sweep volumes compared with [Morph], thus pointing toward a smaller energetic “gap” the lattice needs to accommodate when dealing with the movement and geometric changes in the cation.

In stark contrast, [Morph] exhibits a fundamentally different interaction profile. The quaternary alkyl structure sterically shields the cationic nitrogen and prevents direct anion approach. Consequently, the lattice is forced to compensate through secondary, less energetically favorable interactions. The high enrichment of O···H contacts in [Morph] arises largely from *N*-methyl hydrogens interacting with adjacent ether oxygens on neighboring cations. While these cation–cation interactions provide some local organization, they are geometrically constrained and space-inefficient compared to direct ion pairing. This distinction is crucial: [2-MetThia] and [Mim]­[Br] crystallize (weeks) because they achieve efficient ion pairing, eventually, via the π-face and hydrogen bonds; yet [Morph] persists as a supercooled liquid (years) because its steric alkyl bulk blocks the anion, forcing the system into a frustrated, low-density packing motif dominated by inefficient cation–cation contacts.

### 3.3 The Cost of Sulfur: Energetic Insights from 2-MetThia

*CrystalExplorer* energy framework calculations^57,58^ were used to analyze the relative strength of the interactions in the immediate shell of the [Dime] and [2-MetThia] cations. Only the major portion of the disorder for [2-MetThia] was used for these calculations. Given that [Dime] is, more or less, an ordered ideal within this set of compounds, the insights gained by contrasting these systems sheds light on why [2-MetThia] may exist as a supercooled liquid and the impact of the sulfur moiety on crystallization. Two sets of calculations are shown in Table 3: (1) cation–cation (C^+^–C^+^) energies; (2) cation–anion (C^+^–A^–^) energies.

**Table 3:** Tabulated Energy Components for the Interactions between Cations and Anions in the Immediate Coordination Sphere of the Cation in the Crystal^a^

crystal	label	distance (Å)	*E*_Coulomb_	*E*_dispersion_	*E*_exchange_	*E*_polarization_	*E*_repulsion_	*E*_total_
Cation–Cation Interactions (C^+^–C^+^)								
[2-MetThia]	12	5.37	185.3	–21.9	–28.1	–23.3	50.0	**156.1**
[Dime]	13	5.58	170.8	–21.3	–22.0	–21.2	39.2	**141.5**
[2-MetThia]	11	7.36	172.7	–12.3	–14.0	–16.9	24.8	**152.5**
[Dime]	12	8.50	152.3	–1.7	–0.1	–5.3	0.1	**146.5**
[2-MetThia]	10	10.06	147.1	–4.8	–7.5	–7.7	13.0	**139.0**
[Dime]	11	10.53	145.3	–4.4	–3.1	–7.8	5.1	**135.6**
[Dime]	10	11.89	124.3	–1.6	–0.3	–4.2	0.5	**119.5**
Cation–Anion Interactions (C^+^–A^–^)								
[2-MetThia]	6	4.56	–271.2	–27.5	–33.1	–32.7	59.7	**–310.9**
[Dime]	6	4.23	–274.5	–28.9	–27.4	–34.5	49.5	**–319.1**
[2-MetThia]	5	5.52	–278.9	–21.7	–28.7	–35.6	52.9	**–315.8**
[Dime]	5	5.14	–273.1	–22.5	–24.6	–34.4	45.0	**–311.9**
[2-MetThia]	4	6.36	–223.7	–12.0	–16.1	–22.4	29.2	**–246.5**
[Dime]	4	6.78	–207.9	–11.7	–14.0	–20.2	25.4	**–229.6**
[2-MetThia]	3	6.48	–213.1	–10.3	–10.1	–19.3	18.3	**–234.3**
[Dime]	2	7.34	–169.9	–7.0	–4.1	–9.6	7.7	**–182.5**
[2-MetThia]	2	7.83	–159.1	–9.2	–6.0	–9.6	10.7	**–173.5**
[2-MetThia]	8	8.03	–150.0	–3.4	–1.1	–5.9	2.1	**–157.5**
[Dime]	8	8.06	–151.7	–5.0	–3.1	–6.8	5.8	**–160.5**
[Dime]	3	8.40	–153.4	–9.7	–7.8	–9.2	13.8	**–167.3**
[Dime]	1	8.46	–211.9	–6.6	–10.3	–16.0	18.6	**–226.8**
[2-MetThia]	1	8.50	–143.8	–4.0	–2.5	–5.3	4.7	**–150.7**
[Dime]	9	8.54	–140.8	–3.5	–1.4	–4.9	2.8	**–147.3**
[Dime]	7	8.98	–172.6	–4.6	–2.4	–7.7	4.4	**–182.1**
[2-MetThia]	9	9.28	–167.2	–4.1	–1.3	–6.9	2.7	**–175.8**
[2-MetThia]	7	10.06	–160.0	–2.3	–0.4	–5.6	0.8	**–166.4**

a Interactions are sorted by comparable distance to aid in comparing the two structures. Labels are used for discussion and are from the calculations (see Supporting Information). All energies in kJ mol^–1^.

With respect to the C^+^–C^+^ energies, [2-MetThia] consistently exhibits higher repulsive terms than [Dime] at comparable distances. While the Coulombic term for the [2-MetThia] interaction (Label 12) is predictably higher than [Dime] due to the shorter contact distance (5.37 Å vs 5.58 Å), the primary distinction lies in the repulsion term, which is ∼27% higher than in [Dime]. This trend of elevated repulsion persists through both C^+^–C^+^ and C^+^–A^–^ shells and likely originates from the larger van der Waals radius and diffuse electron cloud of the thiazolium sulfur atom. Conversely, this diffuse density also provides a more potent attractive moiety, evidenced by the consistently higher dispersion and exchange components in [2-MetThia]. As a result, the sulfur atom acts as a source of crystallization frustration: it promotes local stabilization through dispersion and polarization while simultaneously imposing an energetic penalty for close-range packing, likely contributing to the observed supercooling and lattice disorder.

Although both [Dime] and [2-MetThia] exhibit comparable Coulombic stabilization arising from their formally charged ions, the two systems differ markedly in the balance between short-range stabilizing energetic contributions and Pauli repulsion. For the closest C^+^–C^+^ contacts, [Dime] (5.58 Å) exhibits a combined short-range stabilizing contribution (*E*_stab_ = *E*_disp_ + *E*_exch_ + *E*_pol_) of −64.5 kJ mol^–1^, offset by a repulsive term (*E*_Rep_) of +39.2 kJ mol^–1^, yielding a net interaction energy of +141.5 kJ mol^–1^. In contrast, the analogous [2-MetThia] contact at a slightly shorter distance (5.37 Å) benefits from a larger short-range stabilizing contribution of −73.3 kJ mol^–1^; however, this stabilization is counterbalanced by a substantially larger repulsive penalty of +50.0 kJ mol^–1^, resulting in a less favorable net interaction energy of +156.1 kJ mol^–1^.

A similar pattern is observed across the C^+^–A^–^ interaction shell. For the closest C^+^–A^–^ contacts (Label 6), [Dime] exhibits *E*_stab_ = −90.8 kJ mol^–1^ with *E*_Rep_ = +49.5 kJ mol^–1^, whereas the corresponding interaction in [2-MetThia] shows a comparable stabilizing contribution (*E*_stab_ = −93.3 kJ mol^–1^) but a significantly larger repulsive penalty (*E*_Rep_ = +59.7 kJ mol^–1^). Thus, while the stabilizing contribution in [2-MetThia] is only 2.5 kJ mol^–1^ stronger, the associated energetic penalty is increased by 10.2 kJ mol^–1^. Collectively, these results demonstrate that although [2-MetThia] is capable of forming stronger local stabilizing interactions than [Dime], such interactions incur a disproportionately steeper energetic cost at short-range. This imbalance between enhanced short-range attraction and rapidly increasing repulsion restricts the range of geometries that can be simultaneously satisfied within the lattice, providing a quantitative energetic basis for increased packing frustration and offering an energetic-based framework for the pronounced supercooling observed for [2-MetThia].

Beyond the impact of sulfur, both compounds display the same π interaction motifs in the crystal. Specifically, within the [2-MetThia] C^+^–C^+^ interaction shell, Labels 11 and 12 correspond to two π-type contacts: a C4|C5–H···π interaction (Label 11) and a C2–CH_3_···π interaction (Label 12) (Figure 8). These two interactions differ in total energy by only 3.6 kJ mol^–1^. This energetic similarity is particularly noteworthy given the approximately 2 Å difference in their respective contact distances (7.36 Å vs 5.37 Å). Such energetic similarity suggests a packing landscape in which the system can access spatially distinct configurations with minimal energetic cost. This structural freedom inhibits the establishment of a single dominant packing motif necessary for the propagation of long-range order, thereby facilitating the metastable supercooled state and contributing to the observed disorder. The subtle energetic preference for interaction 12 over interaction 11 in [2-MetThia] may further help rationalize the observed major and minor disorder components (ca. 80:20 ratio).

### 3.4 Molecular Volume as a Structural Factor

Building on previously reported studies, we examined the role of π interactions by contrasting benzyl and cyclohexyl substituents.^59,60^ With considerable experimental difficulty, we successfully grew single crystals of the cyclohexyl-substituted [Dime] derivative, [CH_*x*_Dime]. The asymmetric unit of [CH_*x*_Dime] is shown in Figure 10. When considered alongside the structures of [Dime] and [2-MetThia], this system presents a particularly informative data set that not only helps rationalize the observed supercooling, disorder, and ordering behavior, but also hints at a deeper and potentially general structural principle governing benzylated ILs.

**Figure 10:**
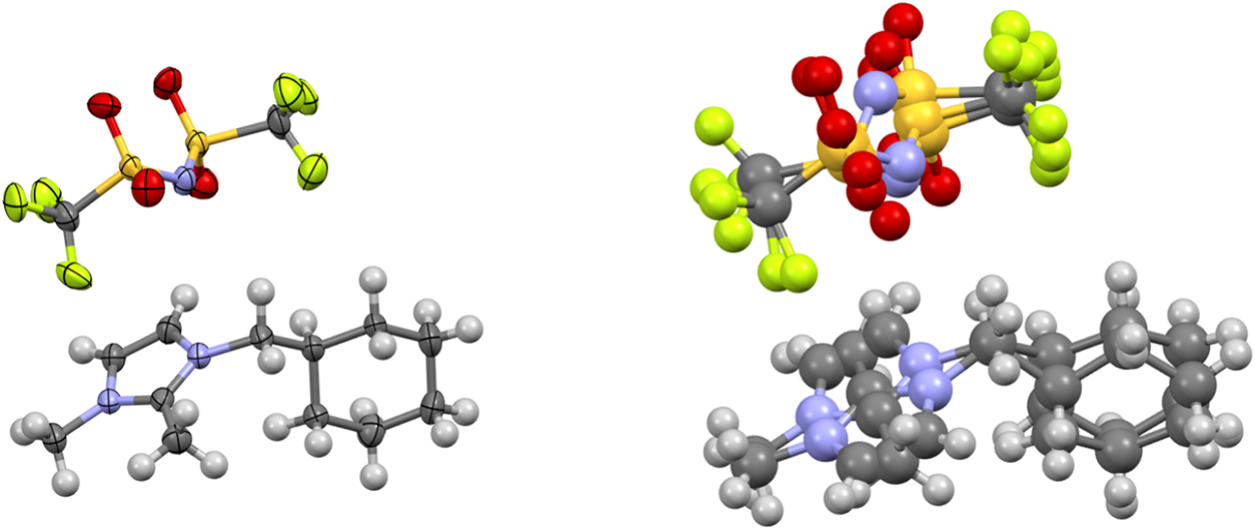
Asymmetric unit of [CHxDime]­[NTf_2_] shown with and without disorder.

Several key structural features emerge from the crystal structure of [CH_*x*_Dime]. Most notably, the cation is disordered through inversion, giving rise to major and minor components though they are of approximately equal occupancy within the crystal. Each disorder component independently occupies a similar static volume, with an average Hirshfeld surface volume of 256.5 Å^3^. However, the total configurational envelope sampled by the disordered cation is larger (274.3 Å^3^), reflecting the expanded spatial region accessed through inversion. A similar disorder motif is observed in [2-MetThia], where ring-inversion of the cationic headgroup likewise generates a two-part model. In [CH_*x*_Dime], inversion of the cyclohexyl group accompanies this process, an effect that would be far more difficult to detect in the aromatic thiazolium analogue.

To better understand the origin of this disorder, we undertook a comparative analysis of the cation volumes. The structure of 1,2-dimethylimidazole was recently reported,^61^ and Hirshfeld surface analysis yields a volume of 132.8 Å^3^ for this simple heterocycle moiety. Although the volume of the corresponding cationic form would be expected to be modestly smaller, this difference is likely minor for the present comparative analysis. Using the cation volumes derived from Hirshfeld surfaces, the benzyl substituent in [Dime] accounts for approximately 123.8 Å^3^, while the methylcyclohexyl group in [CH_*x*_Dime] occupies a nearly identical volume of 123.7 Å^3^. Thus, within the crystalline environment, benzyl and cyclohexyl groups occupy essentially the same volume, an initially counterintuitive result.

It is important to note that these volumes are nondirectional. Simply, identical volumes can arise from substituents with markedly different shapes and spatial distributions. Nonetheless, this isovolumetric relationship allows us to largely decouple substituent size from interaction topology when interpreting the observed disorder. In this context, generic volume-based effects can be reasonably excluded as the primary driver of the differing crystallographic behavior of these systems, allowing us to search for a more complex reason for their structural behavior.

With these considerations in mind, a unifying picture of the observed disorder begins to emerge. The static volume of the [Dime] cation in [Dime] is 256.6 Å^3^, while that of [2-MetThia] is smaller at 241.0 Å^3^. In [2-MetThia], inversion disorder inflates the effective volume sampled by the cation to 251.7 Å^3^, bringing it much closer to that of [Dime]. Structurally, replacing the *N*-methyl group in [Dime] with the sulfur atom of the thiazolium ring reduces the static volume by approximately 15 Å^3^; inversion of the thiazolium heterocycle effectively compensates for this reduction. The persistence of similar π-interaction motifs in both systems (see Section 3.2.2) suggests that these interactions act as anchoring synthons, biasing the structures toward related packing arrangements despite differences in static cation size.

In [CHxDime], the static cation volume is 256.5 Å^3^, placing it squarely within this apparent volumetric “sweet spot.” However, substitution of the π-containing benzyl group with a cyclohexyl moiety eliminates the directional H···π interactions present in [Dime] and [2-MetThia]. While [CHxDime] exhibits a higher fraction of H···H contacts (39.9%, *E*_HH_ = 0.85), these interactions are not statistically favored. Instead, the enrichment ratios reveal a substantial increase in C···F and C···O contacts (*E*_CF_ = 5.03, *E*_CO_ = 1.39), indicating a reorganization of the interaction landscape with respect to both anion and cation contacts.

In both [Dime] and [2-MetThia], the anions preferentially reside above the π systems of the cationic head groups, allowing the central imide nitrogen to engage in close contacts with the formally positive imidazolium nitrogens or thiazolium sulfur. In [CH_*x*_Dime], these preferred positions are sterically less accessible due to the cyclohexyl ring, resulting in a complete suppression of such contacts (*E*_CN_ = 0). Compensation occurs through increased interaction with oxygen and fluorine atoms of the anion. We therefore propose that while benzyl and methylcyclohexyl substituents are isovolumetric in the crystal, differences in their spatial dimensionality sterically prevent optimal anion positioning in [CH_*x*_Dime]. Simply, the anions reside in different orientations and geometries in each system, shifting the interactions (Figure 11). The combined loss of directional CH_3_···π interactions and weakened electrostatic anchoring provides a natural explanation for the enhanced disorder observed in this structure, as well as the difficulty encountered in its crystallization.

**Figure 11:**
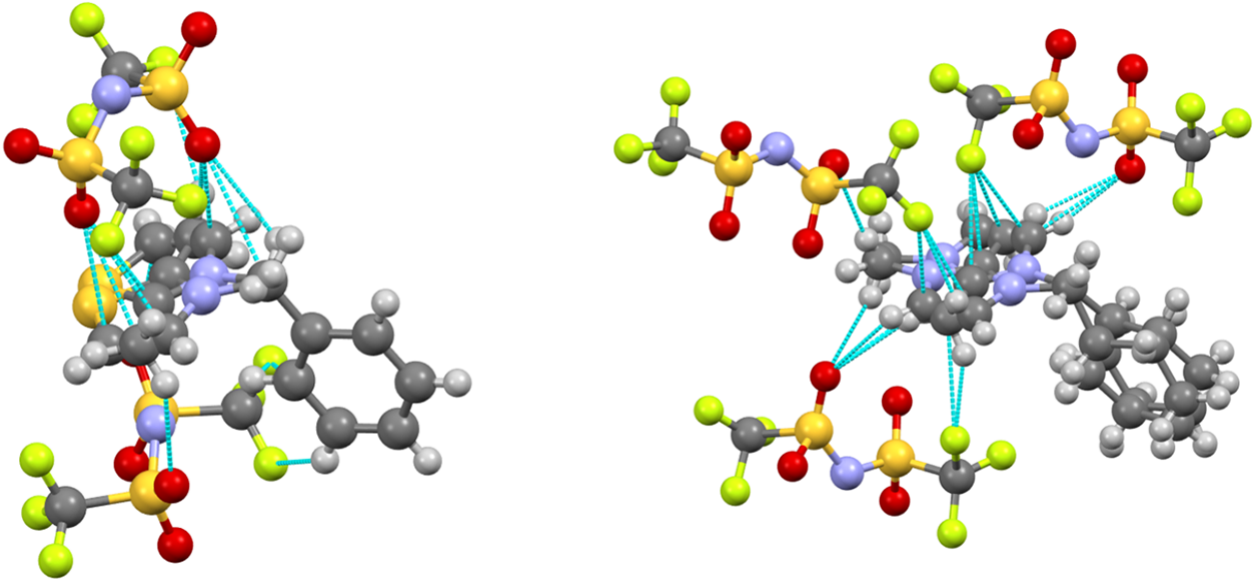
Depiction of the anions in [2-MetThia]­[NTf_2_] and [CH_*x*_Dime]­[NTf_2_]. Changes from the benzyl to cyclohexyl moiety sterically block the formation of anion···π interactions, leading to disorder.

Taken together, these structures reinforce the central role of molecular volume in governing the internal mechanics of ILs, while simultaneously highlighting the limitations of volume as a standalone descriptor. Although the recurrence of cationic volumes near ∼250 Å^3^ across the present systems is noted (the average volume for all cations, disorder included, is 258.46 Å^3^), this observation is best viewed as contextual rather than prescriptive. In each case, comparable effective volumes are achieved through different structural means, namely static packing, conformational flexibility, or disorder. Together this emphasizes that how volume is distributed and accessed is an important consideration, as is absolute magnitude.

These concepts align naturally with volume-based thermodynamics (VBT),^62^ which emphasize molecular volume as a first-order variable influencing cohesive energy, entropy, and transport behavior in ionic systems. From this standpoint, disorder is not merely a crystallographic artifact but represents structural responses that allow ILs to accommodate volume while minimizing energetic penalties. Simply, we offer this analysis as evidence that disorder can function as a meaningful structural metric in understanding molecular design and behavior, rather than merely a crystallographic inconvenience. In other words, the disorder could very well be a result of some larger principle rather than just “appearing” without reason.

As an important contextual point, the cationic volumes reported herein are necessarily derived from the crystalline state and therefore reflect volumes that are compatible with crystallization. Within this data set, a recurring cationic volume of approximately 260 Å^3^ emerges not as an optimal or prescriptive value, but as one that successfully accommodates crystallization. By contrast, benzylated cations whose effective volumes deviate substantially from this metric may be underrepresented precisely because they resist crystallization and persist as liquids. Viewed through this lens, the present results should be interpreted as a set of ‘crystallographic survivorship bias.’ Simply, this value may highlight volumes that more readily crystallize rather than defining the full and definitive design space of benzylated ILs.

### 3.5 ADP Ellipsoids as Structural Maps

Atomic displacement parameters (ADPs), commonly visualized as ellipsoids, provide clues pointing toward both local molecular motion and lattice compliance within the crystal. The structures of [Dime] and [5-NO_2_-Dime] cations were examined through detailed interpretation of their ADPs. Rather than treating ADPs solely as refinement artifacts, deliberate analysis of these parameters provides quantitative insight into molecular motion within the crystal lattice.^43^ In this way, ADP analysis allows us to support several structural hypotheses developed herein, most notably those related to benzyl group motion, while also establishing broader structural principles governing these ILs. The relevant numerical data are summarized in Table 4.

**Table 4:** Average *U*_eq_ Values for the Atoms in the Discrete Moieties within the Cations of [Dime] and [5-NO_2_-Dime]^a^

fragment	atoms	[Dime] Avg U_eq_ (Å^2^)	[Dime] Avg *k*_iso_ (N/m)	[5-NO_2_-Dime] Avg *U*_eq_ (Å^2^)	[5-NO_2_-Dime] Avg *k*_iso_ (N/m)
Imidazolium Ring	N1, C2, N3, C4, C5	0.0152	9.3	0.0205	10.1
Benzyl Linker	C6	0.0163	8.6	0.0223	9.3
Phenyl Ipso	C7	0.0127	11	0.0199	10.4
Phenyl Ortho	C8, 12	0.0161	8.7	0.0266	7.8
Phenyl Meta	C9, 11	0.0198	7.1	0.0347	6.0
Phenyl Para	C10	0.0196	7.1	0.0364	5.7
Methyl (N3)	C13	0.0219	6.4	0.0300	6.9
Methyl (C2)	C14	0.0207	6.7	0.0258	8.0
Nitro Group	N5, O1, O2			0.0327	6.3

a *k*_iso_ values are effective isotropic stiffness parameters used as comparative measures of lattice confinement; larger values indicate more rigid local environments.

To begin, the cations were partitioned into chemically meaningful fragments (methyl groups, benzyl linker, phenyl ring, and imidazolium core), and the average displacement parameters for atoms within each fragment were evaluated. This fragment-averaged approach reveals several clear trends. In both crystal structures, the imidazolium core represents the most rigid component of the cation, exhibiting the lowest average displacement parameters. This behavior is consistent with the role of the imidazolium ring as the primary electrostatic anchor of the system: its delocalized π system engages in strong Coulombic interactions with surrounding anions on both faces of the ring, effectively constraining its motion relative to the more peripheral substituents.

Second, in both systems the methyl groups exhibit significant motion. This behavior is expected for IL systems more broadly, as methyl substituents introduce asymmetry and typically engage only in weaker, largely nondirectional interactions, which permit increased mobility. Comparison of the equivalent displacement parameter of C14 in [Dime] and [5-NO_2_-Dime] reveals a change of approximately 25%, which is notably smaller than the approximately 50% change anticipated based solely on the difference in data collection temperature. This suppressed increase in displacement can be attributed to changes in the local interaction environment. In [Dime], the C14 hydrogens participate in the previously discussed H···π interactions that promote longer-range ordering. In contrast, in [5-NO_2_-Dime] these interactions are replaced by more weakly defined contacts involving symmetry-adjacent aromatic hydrogens on the cation and the benzyl moiety. More importantly, the nitro substituent exerts a strong electronic influence on the imidazolium ring through electron withdrawal, which strengthens anion interactions with the imidazolium π system. As a result, the anions reside closer to the plane of the ring, effectively restricting methyl group motion despite the higher measurement temperature.

Finally, the benzene moiety represents a particularly informative region of the cation. In both systems, the benzene ring displays pronounced liberational motion. A systematic analysis of the aromatic carbons, progressing from the ipso position through the ortho, meta, and para positions, reveals a stepwise increase in atomic displacement parameters. The ipso carbon functions as a local anchor point for the ring, whereas the meta and para positions exhibit substantially larger displacements. This behavior is analogous to that observed for alkyl substituents in ILs, where an increasing distance from the imidazolium core correlates with a greater number of energetically accessible conformations (e.g., the symmetry breaking region^63^). Such enhanced flexibility at the periphery of the cation contributes to disorder and can hinder crystallization.

Examination of the anisotropy ratios (*U*_3_/*U*_1_) at the para positions of the benzyl ring shows that C10 has a lower ratio in [5-NO_2_-Dime] (2.41) than in [Dime] (2.58), despite exhibiting a substantially larger *U*_eq_ value (see S25 and S26). Here, *U*_3_ corresponds to the largest principal axis of the atomic displacement ellipsoid, such that elevated *U*_3_/*U*_1_ ratios indicate directional motion. A plausible explanation for this behavior is that, at elevated temperature, the benzyl moiety undergoes both rocking and twisting motions, resulting in displacement along multiple directions rather than motion dominated by a single principal axis. Such multidimensional motion effectively reduces the observed anisotropy ratio even as the overall displacement amplitude increases. Evidence for this type of behavior is observed upon examination of the φ1 and φ2 torsion angles across the set of compounds studied herein (see Table S12).

Because the [Dime] and [5-NO_2_-Dime] structures were collected at different temperatures, specifically 101 and 150 K respectively, temperature-normalized stiffness parameters (*k*_iso_) were calculated to enable a more direct comparison between the two structures. Several important structural trends emerge from this analysis. First, in both compounds, the imidazolium core represents one of the most rigid regions of the molecule, exhibiting the second highest mean *k*_iso_ value in each case. This observation is consistent with the imidazolium ring functioning as the Coulombic anchor within the lattice, engaging in strong electrostatic interactions with the surrounding anions. Accordingly, the nitrated imidazolium core in [5-NO_2_-Dime] displays a 9.1% higher *k*_iso_ value (9.3 N/m) relative to [Dime] (10.1 N/m). The rationale for this is that the nitro group in [5-NO_2_-Dime] results in a more positive imidazolium ring, thus allowing for shorter contacts with the anion resulting in a more rigid moiety.

Second, the nitro moiety itself appears to be structurally soft, with a summed *k*_iso_ value of 6.3 N/m, similar to methyl groups in the structures. This behavior may arise from the local environment of the nitro group, where portions of the nitro moiety are surrounded by fluorine and oxygen atoms from the [NTf_2_]^−^ anion, resulting in a relatively shallow effective potential well.

Finally, both systems exhibit the same systematic trend within the benzyl moiety, characterized by decreasing *k*_iso_ values upon progression from the ortho to the meta to the para positions of the aromatic ring. In both series, the *ipso* carbon serves as the most structurally robust position, followed by a significant reduction in stiffness at the ortho and meta carbons, corresponding to an approximate 20% decrease in each case. In [Dime], the *para* carbon shows a very small decrease in stiffness relative to the meta position, whereas in [5-NO_2_-Dime] the *para* carbon continues the expected decrease. This divergence is likely influenced by differences in torsion angles and benzyl conformations adopted in the two structures.

In summary, analysis of the atomic displacement parameters provides insight into the structural dynamics and mechanical behavior of these ionic liquids. Regions characterized by lower *k*_iso_ values correspond to compliant sites that are more susceptible to thermally induced structural changes. Notably, the trends discussed are systematic across chemically related positions and fragments, supporting a dynamic rather than static origin for the observed anisotropy. The concentration of low-stiffness regions within the benzyl and nitro-substituted portions of the cation suggests that these fragments act as points of mechanical flexibility within the lattice, rendering them particularly sensitive to thermal stress and positioning them as likely contributors to temperature-driven structural transformations.

### 3.6 Summary of Structural Principles

Taken together, these structures suggest several broader principles for benzylated ILs which we attempt to summarize in Table 5. First, supercooling does not arise from a single structural feature, but from the combined effects of conformational flexibility, competing local packing motifs, and steric features that hinder efficient propagation of long-range order. Second, crystallographic disorder is not merely a refinement complication but can serve as a structural indicator of frustrated packing and accessible alternative arrangements within the lattice. Third, changes in cation architecture, whether electronic, steric, or conformational, shift how volume and intermolecular contacts are accommodated, thereby influencing whether a given system crystallizes directly, crystallizes only after long-lived supercooling, or tolerates disorder in the solid state. In this sense, the set of structures reported herein provides not a single design rule, but a comparative framework for understanding how IL structure relates to crystallinity.

**Table 5:** Summary of the Key Crystallization Behavior, Disorder Features, and Structural Implications for the Ionic Liquids Examined in This Study

compound	crystallization behavior	disorder/modulation	key structural feature	main takeaway
[Dime][NTf_2_]	Direct crystallization	Ordered	Benzylated ordered reference; CH_3_···π motifs	Reference for comparison of π-mediated packing
[5-NO_2_-Dime][NTf_2_]	Direct crystallization	Ordered	Nitro substitution redistributes preferred contacts. Changes in benzyl torsion angle.	Electronic changes shifts interaction preferences, changing torsion angles
[2-MetThia][NTf_2_]	Crystallized from supercooled melt	Cation disorder; twinning	Preserved CH_3_···π interactions via disordering.	Supercooling linked to frustrated but still accessible ordering via sulfur moiety
[Morph][NTf_2_]	Crystallized from long-lived supercooled melt	Cation/anion disorder; commensurate modulation	Large configurational envelope and modulation	Extreme packing frustration delays crystallization
[Mim][Br]	Previously reported supercooled system	Two independent cations, distinct torsion angles	H-bonding plus competing π preferences	Contrasting system showing alternate route to frustrated packing via competitive interactions
[CHxDime][NTf_2_]	Crystallized with disorder	Multiple disorder in cation/anion	Nonaromatic, isovolumetric contrast to benzyl systems	Separates steric/volumetric effects from π-mediated packing

## 4 Conclusions

Benzylated ILs remain an underexplored subclass of task-specific ILs, incompletely characterized despite their structural complexity. Although early reports generated significant interest, these materials have largely been overshadowed by alkylated analogues, in part due to their higher melting points and viscosities. In the present study, however, these same features proved advantageous, enabling a detailed examination of structural design principles through crystallographic and computational analysis.

Three benzylated cations examined herein crystallized from long-lived supercooled melts. Across these systems, supercooling is consistently associated with multiple accessible conformations, comparable local interaction environments, degeneracy among key packing motifs such as CH_3_···π contacts, and steric features that hinder efficient packing. These factors act together to frustrate the development of long-range order and thereby delay crystallization. In this sense, there is no single hierarchical explanation for the formation of these supercooled liquids; rather, their behavior emerges from the combined effects of disorder, conformational flexibility, interaction degeneracy, and steric blocking.

Across the benzylated ILs examined here, effective cationic volume (∼260 Å^3^), achieved through static packing, conformational flexibility, or crystallographic disorder, emerges as a meaningful and unifying structural descriptor. Rather than identifying a single optimal volume, this analysis demonstrates that the manner in which volume is accommodated within the lattice plays a decisive role in shaping intermolecular interactions, promoting or suppressing disorder, and governing crystallization behavior. These observations provide empirical support for volume-based considerations as a practical framework for the rational design of ILs.

Careful analysis of disorder, coupled with rigorous geometric modeling, reveals that disorder in these systems is not incidental but mechanistically informative. In the [Morph] structure, liberational motion of the benzyl moieties manifests as two-part disorder, revealing competing local energy minima that define a structural pathway toward supercooling, particularly when considered alongside translational motion of the cationic core. In contrast, the ring-inversion disorder observed in [2-MetThia] implicates a similar frustration mechanism, albeit one operating on a substantially shorter time scale. In both cases, disorder provides structural evidence for competing packing motifs in the solid state.

More broadly, this study demonstrates that crystallographic observables—including effective volume, disorder parameters, and enrichment ratios—when interpreted as structural descriptors rather than static measurements, can be leveraged to rationalize the design of novel ILs. By integrating volume-based concepts, disorder analysis, and interaction statistics, this work underscores the prospective value of solid-state structure in IL design, positioning crystallographic analysis as a useful guide rather than merely a retrospective characterization tool.

## Supplementary Material



## Notes

Hirshfeld surface enrichment ratios and contact percentages; geometric swept-volume calculations and kinematic analysis; crystallographic information tables; CrystalExplorer interaction energies; molecular structure data; Hirshfeld surface percentage tables; PLATON-derived atomic displacement parameters; NMR spectra (PDF)

## Abbreviations

Bmim: 1-butyl-3-methylimidazolium
BzMim: 3-benzyl-1-methylimidazolium
Dime: 3-benzyl-1,2-dimethylimidazolium
Morph: 4-benzyl-4-methyl-4-morpholinium
2-MetThia: 3-benzyl-2-methyl-1,3-thiazol-3-ium
5-NO_2_-Dime: 3-benzyl-1,2-dimethyl-5-nitro-3-imidazolium
CH_*x*_Dime: 3-(cyclohexylmethyl)-1,2-dimethyl-3-imidazolium
NTf_2_: bis­(trifluoromethanesulfonyl)­imide
