# Supplementary material for: Ordo ab Chao: Crystallographic Disorder as a Window into Ionic Liquid Structure
Source: Cryst Growth Des. 2026 Jun 2;26(12):4612–28. doi: 10.1021/acs.cgd.6c00247 (PMC13281378; doi:10.1021/acs.cgd.6c00247)
Supplement: Supplementary file 1 [file cg6c00247_si_001.pdf]

Ordo ab Chao: Crystallographic Disorder as a Window into Ionic Liquid Structure

Joseph Cooper[a], Marija Scheuren[a], Lara I. Teodoro[a], Reychell A. Jerdo[b], Kylie M. Allen[b], Christopher M. Butch[b], Mariana E. Toner[b], Matthias Zeller[c], Arsalan Mirjafari[b,*], and Patrick C. Hillesheim[d,*]

[a]Department of Chemistry and Physics, Ave Maria University, Ave Maria, Florida 34142, United States
[b]Department of Chemistry, State University of New York at Oswego, Oswego, New York 13126, United States
[c]Department of Chemistry, Purdue University, West Lafayette, Indiana 47907, United States
[d]Department of Chemistry, Illinois State University, Normal, Illinois 61761, United States
[*]Corresponding authors: Arsalan.Mirjafari@oswego.edu; pchille@ilstu.edu

Contents

1 Hirshfeld Surface Enrichment Ratios S3
1.1 Dime[NTf_2] . . . S3
1.2 $5NO_2$Dime[NTf_2] . . . S3
1.3 2MetThia[NTf_2] . . . S4
1.4 CHxDime[NTf_2] . . . S4
1.5 Dime[I] . . . S5
1.6 MimBr . . . S6
1.7 Morph . . . S7

2 Geometric Calculation of Static/Swept Volumes and Kinematics S9
2.1 Calculation of Swept Volumes for Disordered Moieties . . . S9
2.2 Rigid-Body Swept Volume Calculation for Benzyl Ring Motion in MimBr . . . S14

3 Crystallographic Information Tables S17

4 CrystalExplorer Interaction Energies S18
4.1 Dime[NTf_2] . . . S18
4.2 2MetThia[NTf_2] . . . S18

5 Molecular Structure Data S20

6 Hirshfeld Surface Percentage Tables S21
6.1 MimBr . . . S21
6.2 $5NO_2$Dime[NTf_2] . . . S21

6.3 Dime[NTf_2] . . . S22
6.4 Dime[I] . . . S23
6.5 2MetThia[NTf_2] . . . S23
6.6 Morph[NTf_2] . . . S23
6.7 CHxDime[NTf_2] . . . S24

7 Atomic Displacement Parameters (PLATON) S25
7.1 Dime[NTf_2] Dataset . . . S25
7.2 5-NO_2-Dime[NTf_2] Dataset . . . S25

8 NMR Spectra S27
8.1 Dime[NTf_2] . . . S28
8.2 5-NO_2-Dime[NTf_2] . . . S29
8.3 2-MetThia[NTf_2] . . . S30
8.4 Morph[NTf_2] . . . S31
8.5 CHxDime[NTf_2] . . . S32

1 Hirshfeld Surface Enrichment Ratios

The following tables provide the Hirshfeld Surface Enrichment Ratios (E_{XY}), along with the Actual Contact Percentage (C_{XY}) and Random Contact Percentage (R_{XY}) for the compounds studied.

1.1 Dime[NTf_2]

Table S1: Hirshfeld surface enrichment ratios for Dime[NTf_2].

Interaction	Actual % (C_{XY})	Random % (R_{XY})	Ratio (E_{XY})
H...H	17.9	31.36	0.57
H...C	16.3	11.31	1.44
H...N	2.6	3.02	0.86
H...O	21.6	13.89	1.56
H...F	35.5	21.06	1.69
H...S	0.2	0.11	1.79
C...C	0.0	1.02	0.00
C...N	0.1	0.55	0.18
C...O	2.0	2.50	0.80
C...F	1.8	3.80	0.47
C...S	0.0	0.02	0.00
N...N	0.6	0.07	8.23
N...O	1.2	0.67	1.79
N...F	0.3	1.02	0.30
N...S	0.0	0.01	0.00
O...O	0.0	1.54	0.00
O...F	0.0	4.66	0.00
O...S	0.0	0.02	0.00
F...F	0.0	3.53	0.00
F...S	0.0	0.04	0.00
S...S	0.0	0.00	0.00

1.2 $5NO_2$Dime[NTf_2]

Table S2: Hirshfeld surface enrichment ratios for $5NO_2$Dime[NTf_2].

Interaction	Actual % (C_{XY})	Random % (R_{XY})	Ratio (E_{XY})
H...H	18.0	25.91	0.69
H...C	2.7	6.01	0.45
H...N	1.9	3.61	0.53
H...O	41.1	24.74	1.66
H...F	20.0	15.58	1.28
H...S	0.1	0.05	1.96
C...C	0.0	0.35	0.00
C...N	0.9	0.42	2.15

Table S2: Hirshfeld surface enrichment ratios for $5NO_2Dime[NTf_2]$.

**Interaction**	**Actual % (C_{XY})**	**Random % (R_{XY})**	**Ratio (E_{XY})**
C...O	1.4	2.87	0.49
C...F	6.8	1.81	3.77
C...S	0.0	0.01	0.00
N...N	0.9	0.13	7.14
N...O	1.2	1.73	0.70
N...F	1.3	1.09	1.20
N...S	0.0	0.00	0.00
O...O	1.2	5.90	0.20
O...F	2.5	7.44	0.34
O...S	0.0	0.02	0.00
F...F	0.0	2.34	0.00
F...S	0.0	0.02	0.00
S...S	0.0	0.00	0.00

1.3 2MetThia[NTf_2]

Table S3: Hirshfeld surface enrichment ratios for 2MetThia[NTf_2].

**Interaction**	**Actual % (C_{XY})**	**Random % (R_{XY})**	**Ratio (E_{XY})**
H...H	11.5	25.55	0.45
H...C	16.7	9.60	1.74
H...N	2.0	2.07	0.96
H...O	20.1	11.93	1.68
H...F	33.1	19.51	1.70
H...S	6.2	6.87	0.90
C...C	0.0	0.90	0.00
C...N	0.1	0.39	0.26
C...O	1.5	2.24	0.67
C...F	0.7	3.67	0.19
C...S	0.0	1.29	0.00
N...N	0.0	0.04	0.00
N...O	0.7	0.48	1.45
N...F	0.0	0.79	0.00
N...S	1.3	0.28	4.66
O...O	0.0	1.39	0.00
O...F	0.0	4.55	0.00
O...S	1.3	1.60	0.81
F...F	0.0	3.72	0.00
F...S	4.8	2.62	1.83
S...S	0.0	0.46	0.00

1.4 CHxDime[NTf_2]

Table S4: Hirshfeld surface enrichment ratios for CHxDime[NTf_2].

**Interaction**	**Actual %** (C_{XY})	**Random %** (R_{XY})	**Ratio** (E_{XY})
H...H	39.9	47.06	0.85
H...C	0.0	0.75	0.00
H...N	1.4	2.06	0.68
H...O	25.2	17.97	1.40
H...F	30.8	22.30	1.38
H...S	0.0	0.00	0.00
C...C	0.0	0.00	0.00
C...N	0.0	0.02	0.00
C...O	0.2	0.14	1.39
C...F	0.9	0.18	5.03
C...S	0.0	0.00	0.00
N...N	0.0	0.02	0.00
N...O	0.8	0.39	2.04
N...F	0.8	0.49	1.64
N...S	0.0	0.00	0.00
O...O	0.0	1.72	0.00
O...F	0.0	4.26	0.00
O...S	0.0	0.00	0.00
F...F	0.0	2.64	0.00
F...S	0.0	0.00	0.00
S...S	0.0	0.00	0.00

1.5 Dime[I]

Table S5: Hirshfeld surface enrichment ratios for Dime[I].

**Interaction**	**Actual %** (C_{XY})	**Random %** (R_{XY})	**Ratio** (E_{XY})
H...H	64.8	61.23	1.06
H...C	13.4	21.83	0.61
H...N	3.0	3.99	0.75
H...I	10.5	8.22	1.28
C...C	6.2	1.95	3.19
C...N	2.1	0.71	2.95
C...I	0.0	1.46	0.00
N...N	0.0	0.07	0.00
N...I	0.0	0.27	0.00
I...I	0.0	0.28	0.00

1.6 MimBr

Cation 1

Table S6: Hirshfeld surface enrichment ratios for MimBr (Cation 1).

Interaction	Actual % (C_{XY})	Random % (R_{XY})	Ratio (E_{XY})
H...H	60.1	60.22	1.00
H...C	20.2	21.42	0.94
H...N	3.2	3.96	0.81
H...Br	11.6	9.39	1.24
C...C	2.6	1.90	1.37
C...N	1.8	0.70	2.56
C...Br	0.4	1.67	0.24
N...N	0.0	0.07	0.00
N...Br	0.1	0.31	0.32
Br...Br	0.0	0.37	0.00

Cation 2

Table S7: Hirshfeld surface enrichment ratios for MimBr (Cation 2).

Interaction	Actual % (C_{XY})	Random % (R_{XY})	Ratio (E_{XY})
H...H	58.0	59.06	0.98
H...C	21.3	21.67	0.98
H...N	3.0	3.54	0.85
H...Br	13.4	10.37	1.29
C...C	2.6	1.99	1.31
C...N	1.6	0.65	2.47
C...Br	0.1	1.90	0.05
N...N	0.0	0.05	0.00
N...Br	0.0	0.31	0.00
Br...Br	0.0	0.46	0.00

1.7 Morph

Cation 1

Table S8: Hirshfeld surface enrichment ratios for Morph (Cation 1).

**Interaction**	**Actual % (C_{XY})**	**Random % (R_{XY})**	**Ratio (E_{XY})**
H...H	23.6	38.01	0.62
H...C	13.7	8.63	1.59
H...N	2.0	1.23	1.62
H...O	31.9	19.85	1.61
H...F	28.5	17.57	1.62
H...S	0.0	0.00	0.00
C...C	0.0	0.49	0.00
C...N	0.0	0.14	0.00
C...O	0.3	2.25	0.13
C...F	0.0	2.00	0.00
C...S	0.0	0.00	0.00
N...N	0.0	0.01	0.00
N...O	0.0	0.32	0.00
N...F	0.0	0.28	0.00
N...S	0.0	0.00	0.00
O...O	0.0	2.59	0.00
O...F	0.0	4.59	0.00
O...S	0.0	0.00	0.00
F...F	0.0	2.03	0.00
F...S	0.0	0.00	0.00
S...S	0.0	0.00	0.00

Cation 2

Table S9: Hirshfeld surface enrichment ratios for Morph (Cation 2).

**Interaction**	**Actual % (C_{XY})**	**Random % (R_{XY})**	**Ratio (E_{XY})**
H...H	23.8	38.19	0.62
H...C	13.7	8.53	1.61
H...N	3.0	1.85	1.62
H...O	29.7	18.42	1.61
H...F	29.6	18.29	1.62
H...S	0.0	0.00	0.00
C...C	0.0	0.48	0.00
C...N	0.0	0.21	0.00
C...O	0.1	2.06	0.05
C...F	0.0	2.04	0.00
C...S	0.0	0.00	0.00
N...N	0.0	0.02	0.00

Table S9: Hirshfeld surface enrichment ratios for Morph (Cation 2).

**Interaction**	**Actual %** (C_{XY})	**Random %** (R_{XY})	**Ratio** (E_{XY})
N...O	0.0	0.45	0.00
N...F	0.0	0.44	0.00
N...S	0.0	0.00	0.00
O...O	0.0	2.22	0.00
O...F	0.0	4.41	0.00
O...S	0.0	0.00	0.00
F...F	0.0	2.19	0.00
F...S	0.0	0.00	0.00
S...S	0.0	0.00	0.00

Cation 3

Table S10: Hirshfeld surface enrichment ratios for Morph (Cation 3).

**Interaction**	**Actual %** (C_{XY})	**Random %** (R_{XY})	**Ratio** (E_{XY})
H...H	24.1	38.19	0.63
H...C	13.4	8.59	1.56
H...N	2.1	1.30	1.62
H...O	31.8	19.90	1.60
H...F	28.1	17.43	1.61
H...S	0.0	0.00	0.00
C...C	0.0	0.48	0.00
C...N	0.0	0.15	0.00
C...O	0.4	2.24	0.18
C...F	0.1	1.96	0.05
C...S	0.0	0.00	0.00
N...N	0.0	0.01	0.00
N...O	0.0	0.34	0.00
N...F	0.0	0.30	0.00
N...S	0.0	0.00	0.00
O...O	0.0	2.59	0.00
O...F	0.0	4.54	0.00
O...S	0.0	0.00	0.00
F...F	0.0	1.99	0.00
F...S	0.0	0.00	0.00
S...S	0.0	0.00	0.00

2 Geometric Calculation of Static/Swept Volumes and Kinematics

2.1 Calculation of Swept Volumes for Disordered Moieties

To quantify the effective steric bulk of the disordered/modulated ions, we developed a custom Python script to calculate the "swept volume," defined as the spatial union of the van der Waals surfaces occupied by the molecule across its modulation or disorder range.

Input coordinates for the discrete molecular states (e.g., State 1, State 2, State 3) were extracted directly from the CIF. To reconstruct the continuous spatial distribution between these discrete states, we generated a high-density trajectory (typically 20 frames per transition) using a geometry-preserving interpolation scheme:

- **Atom Mapping:** The Hungarian algorithm (linear sum assignment) was employed to match atoms between states by minimizing the total displacement distance. This ensures that rotationally symmetric groups are mapped to their nearest physical neighbors, preventing unphysical atom swapping.
- **Spherical Interpolation:** To prevent bond shortening artifacts during rotation, "riding" atoms were interpolated using Spherical Linear Interpolation (SLERP) relative to their parent heavy atoms. This constraint preserves bond lengths and local bond angles throughout the trajectory.
- **Volume Integration:** The swept volume was determined via Monte Carlo integration. A rectangular bounding box encompassing all generated trajectory frames was sampled using 5×10^6 uniformly distributed random points. A point was classified as occupied if it fell within the Bondi van der Waals radius of any atom in any trajectory frame.

The swept volume is therefore the union of all instantaneous molecular van der Waals volumes sampled along the interpolation pathway. This increase quantitatively accounts for the movement of the benzyl ring and the tilting of the morpholine core as the molecule propagates along the modulation vector.

```
import numpy as np
import re
import os
import sys
import argparse
from scipy.optimize import linear_sum_assignment
from scipy.sparse import csgraph, csr_matrix
from scipy.spatial.distance import pdist, squareform

# ==========================================================================
# CONFIGURATION
# ==========================================================================
DEFAULT_MC_SAMPLES = 5000000    # Publication
DEFAULT_INTERP_FRAMES = 20
GRID_PADDING = 0.5
BOND_CUTOFF = 2.1
# Define which atoms are "Riders" (maintain distance to parent)
# For NTf2, F rides on C, O rides on S.
RIDING_ELEMENTS = ['H', 'F', 'O']
VDW_RADII = {
    'H': 1.20, 'C': 1.70, 'N': 1.55, 'O': 1.52,
    'S': 1.80, 'F': 1.47, 'Cl': 1.75, 'Br': 1.85, 'I': 1.98
```

```python
}

def clean_val(x):
    return float(re.sub(r'\(\d+\)', '', x))

def parse_cif(filename):
    with open(filename, 'r') as f:
        lines = f.readlines()
    cell = {'a': 0.0, 'b': 0.0, 'c': 0.0, 'beta': 90.0}
    atoms = []
    headers = []
    in_loop = False
    for line in lines:
        line = line.strip()
        if not line: continue
        if line.startswith('_cell_length_a'): cell['a'] = clean_val(line.split()[1])
        if line.startswith('_cell_length_b'): cell['b'] = clean_val(line.split()[1])
        if line.startswith('_cell_length_c'): cell['c'] = clean_val(line.split()[1])
        if line.startswith('_cell_angle_beta'): cell['beta'] = clean_val(line.split()[1])
        if line.startswith('loop_'):
            in_loop = False; continue
        if line.startswith('_atom_site_label'):
            in_loop = True; headers = ['_atom_site_label']; continue
        if in_loop and line.startswith('_atom_site_'):
            headers.append(line.split()[0]); continue
        if in_loop and not line.startswith('_'):
            parts = line.split()
            if len(parts) >= len(headers):
                d = {h: parts[i] for i, h in enumerate(headers)}
                if '_atom_site_type_symbol' not in d:
                    d['_atom_site_type_symbol'] = re.sub(r'\d.*', '', d['_atom_site_label'])
                atoms.append(d)
    return cell, atoms

def fractional_to_cartesian(xf, yf, zf, cell):
    a, b, c = cell['a'], cell['b'], cell['c']
    beta_rad = np.radians(cell['beta'])
    x = xf * a + zf * c * np.cos(beta_rad)
    y = yf * b
    z = zf * c * np.sin(beta_rad)
    return np.array([x, y, z], dtype=float)

def get_target_molecule(atoms_subset, target_type='cation'):
    if not atoms_subset: return []
    coords = np.array([a['coords'] for a in atoms_subset])
    elements = [a['element'] for a in atoms_subset]
    dist_matrix = squareform(pdist(coords))
    adj = dist_matrix < BOND_CUTOFF
    n_components, labels = csgraph.connected_components(csgraph=csr_matrix(adj), directed=False, return_labels=True)
    candidates = []
    for i in range(n_components):
        idxs = np.where(labels == i)[0]
        cluster_elements = set([elements[j] for j in idxs])
```

```python
        has_SF = ('S' in cluster_elements) or ('F' in cluster_elements)
        if target_type == 'anion':
            if has_SF: candidates.append(idxs)
        else:
            if not has_SF and ('C' in cluster_elements): candidates.append(idxs)
    if not candidates: return []
    return [atoms_subset[i] for i in max(candidates, key=len)]

def extract_state(atoms_raw, cell, suffix, lattice_shift, keep_disorder_group=None, target='cation'):
    candidates = []
    for d in atoms_raw:
        if not d['_atom_site_label'].endswith(suffix): continue
        grp = d.get('_atom_site_disorder_group', '.')
        if keep_disorder_group is not None:
            if grp != '.' and grp != str(keep_disorder_group): continue
        xf = clean_val(d['_atom_site_fract_x']) + lattice_shift
        yf = clean_val(d['_atom_site_fract_y'])
        zf = clean_val(d['_atom_site_fract_z'])
        d['coords'] = fractional_to_cartesian(xf, yf, zf, cell)
        d['element'] = d.get('_atom_site_type_symbol', '')
        candidates.append(d)
    target_atoms = get_target_molecule(candidates, target_type=target)
    if not target_atoms: return np.array([]), np.array([]), []
    coords = np.array([a['coords'] for a in target_atoms])
    elements = [a['element'] for a in target_atoms]
    radii = np.array([VDW_RADII.get(el, 1.5) for el in elements])
    # Sort for consistent matching (S, F, O, N, C, H)
    combined = sorted(zip(coords, elements, radii), key=lambda x: {'S':0,'F':1,'O':2,'N':3,'C':4,'H':5}.get(x[1], 6))
    return np.array([x[0] for x in combined]), np.array([x[2] for x in combined]), [x[1] for x in combined]

def match_atoms(coords_start, coords_end, elements):
    indices = np.arange(len(elements))
    new_end = np.zeros_like(coords_end)
    for el in set(elements):
        mask = np.array([e == el for e in elements])
        if not np.any(mask): continue
        idx = indices[mask]
        d2 = np.sum((coords_start[idx][:,None,:] - coords_end[idx][None,:,:])**2, axis=2)
        r, c = linear_sum_assignment(d2)
        new_end[idx[r]] = coords_end[idx[c]]
    return new_end

def slerp(v0, v1, t):
    dot = np.clip(np.sum(v0*v1), -1.0, 1.0)
    if dot > 0.9995: return v0 + t*(v1-v0)
    theta = np.arccos(dot)
    return (np.sin((1-t)*theta)/np.sin(theta))*v0 + (np.sin(t*theta)/np.sin(theta))*v1

def interpolate(c1, c2, elements, frames):
    traj = []
    # Identify Riders (F, O, H) and their Parents (C, S, etc.)
    rider_idx = [i for i, x in enumerate(elements) if x in RIDING_ELEMENTS]
    heavy_idx = [i for i, x in enumerate(elements) if x not in RIDING_ELEMENTS]
    parents, d_ref, u1, u2 = {}, {}, {}, {}
```

```python
    if rider_idx and heavy_idx:
        for r in rider_idx:
            # Find nearest non-rider atom (Parent)
            d = np.linalg.norm(c1[heavy_idx]-c1[r], axis=1)
            p = heavy_idx[np.argmin(d)]
            parents[r], d_ref[r] = p, d[np.argmin(d)]
            v1, v2 = c1[r]-c1[p], c2[r]-c2[p]
            u1[r], u2[r] = v1/np.linalg.norm(v1), v2/np.linalg.norm(v2)
    for i in range(frames):
        a = i/(frames-1)
        # Linear interp for parents/heavy atoms
        c = (1-a)*c1 + a*c2
        # SLERP for riders (F, O) around parents
        for r in rider_idx:
            if r in parents:
                c[r] = c[parents[r]] + slerp(u1[r], u2[r], a)*d_ref[r]
        traj.append(c)
    return traj

def mc_vol(coords, radii, n_samples):
    all_c, all_r = np.vstack(coords), np.hstack(radii)
    min_b = np.min(all_c - all_r[:,None], axis=0) - GRID_PADDING
    max_b = np.max(all_c + all_r[:,None], axis=0) + GRID_PADDING
    rng = np.random.default_rng()
    hits = 0; chunk = 50000
    print(f"  MC Sampling ({n_samples} pts)...")
    for i in range((n_samples+chunk-1)//chunk):
        pts = rng.uniform(min_b, max_b, size=(min(chunk, n_samples-i*chunk), 3))
        mask = np.zeros(len(pts), dtype=bool)
        for c, r in zip(coords, radii):
            mask |= np.any(np.sum((pts[:,None,:] - c[None,:,:])**2, axis=2) < r**2, axis=1)
        hits += np.sum(mask)
    return (hits/n_samples) * np.prod(max_b-min_b)

def main():
    p = argparse.ArgumentParser()
    p.add_argument("cif"); p.add_argument("--target", default='cation')
    p.add_argument("-q", "--q1", type=float, default=0.0)
    p.add_argument("-q2", "--q2", type=float, default=-0.3333)
    p.add_argument("-q3", "--q3", type=float, default=-0.6666)
    p.add_argument("-n", "--samples", type=int, default=DEFAULT_MC_SAMPLES)
    a = p.parse_args()
    print(f"--- Processing {a.target.upper()} (Riding F/O Fix) ---")
    cell, atoms = parse_cif(a.cif)
    c1, r1, e1 = extract_state(atoms, cell, '_1', a.q1, 1, a.target)
    c2, r2, e2 = extract_state(atoms, cell, '_2', a.q2, 1, a.target)
    c3, r3, e3 = extract_state(atoms, cell, '_3', a.q3, 1, a.target)
    if not len(c1): sys.exit("Error: No atoms found.")
    # Sort atoms
    c2 = match_atoms(c1, c2, e1)
    c3 = match_atoms(c2, c3, e1)
    cent = np.mean(c1, axis=0)
    c1-=cent; c2-=cent; c3-=cent
    traj = interpolate(c1,c2,e1,20) + interpolate(c2,c3,e1,20) + interpolate(c3,c1,e1,20)
    v_stat = mc_vol([c1], [r1], a.samples)
    v_swep = mc_vol(traj, [r1]*len(traj), a.samples)
    print(f"Static: {v_stat:.2f} A^3")
```

```python
    print(f"Swept:  {v_swep:.2f} A^3")
    print(f"Increase: {((v_swep-v_stat)/v_stat)*100:.1f}%")
    out = a.cif.replace(".cif", f"_{a.target}_FIXED.xyz")
    with open(out,'w') as f:
        for i, fr in enumerate(traj):
            f.write(f"{len(fr)}\nFrame {i}\n")
            for j, x in enumerate(fr): f.write(f"{el[j]} {x[0]:.4f} {x[1]:.4f} {x[2]:.4f}\n")
    print(f"Saved {out}")

if __name__ == "__main__": main()
```

Listing 1: Swept Volume Calculation Script

2.2 Rigid-Body Swept Volume Calculation for Benzyl Ring Motion in MimBr

To quantify the steric volume associated with large-amplitude benzyl ring motion, a rigid-body kinematic model was implemented to calculate the interpolated swept van der Waals volume of benzyl portion of the cation, as inferred from the crystal structures.

Two experimentally resolved conformations of the cation were obtained from an overlay of crystallographic coordinates. The molecule was partitioned into two rigid fragments: (i) the methylimidazolium core including methyl and linker atoms, and (ii) the benzyl aromatic ring. Internal geometries within each fragment were preserved throughout the trajectory.

For each fragment, the optimal rigid transformation between the start and end conformations was determined using a least-squares Kabsch alignment. This yields a rotation and translation that map one conformation onto the other without internal distortion. Intermediate structures were generated by linearly interpolating the fragment centroids while simultaneously interpolating the rotation using scaled rotation vectors, producing a smooth rigid-body trajectory (40 frames).

The swept volume was calculated using Monte Carlo integration. A bounding box enclosing all frames of the trajectory was sampled with 1×10^6 random points. A point was considered occupied if it lay within the Bondi van der Waals radius of any atom in any frame. The swept volume therefore represents the spatial union of all sterically occupied regions during the rigid-body motion of the benzyl ring relative to the cation core.

For comparison, a static reference volume was calculated from the initial conformer using the same Monte Carlo procedure. The percent increase in volume provides a quantitative measure of the additional steric space required to accommodate benzyl ring libration within the crystal lattice.

```python
import numpy as np
from scipy.spatial.transform import Rotation as R
# ==========================================
# CONFIGURATION
# ==========================================
INPUT_FILE = "Overlayed MimBr Cations.xyz"
OUTPUT_FILE = "MimBr_DualRigid_Movie.xyz"
FRAMES = 40                 # Smoothness of the sweep
ATOMS_PER_CATION = 26
MC_SAMPLES = 1000000        # Precision of volume calc (1M is good)
GRID_PADDING = 0.5          # Buffer around the molecule
# Atom Definitions (0-based indices)
# Body 1: Core + Methyl + Linker (Atoms 0-14)
BODY1_INDICES = list(range(0, 15))
# Body 2: Benzene Ring + Ipso Carbon (Atoms 15-25)
BODY2_INDICES = list(range(15, 26))
# VdW Radii (Bondi)
RADII = {
    'H': 1.20, 'C': 1.70, 'N': 1.55, 'Br': 1.85,
    'O': 1.52, 'F': 1.47, 'S': 1.80, 'I': 1.98, 'Cl': 1.75
}

# ==========================================
# 1. HELPER FUNCTIONS
# ==========================================
def read_xyz(filename):
    with open(filename, 'r') as f:
        lines = [l.strip() for l in f.readlines() if l.strip()]
    atom_lines = lines[2:]
    c1, c2 = [], []
    elements = []
    for i, line in enumerate(atom_lines):
```

```python
        parts = line.split()
        if i < ATOMS_PER_CATION:
            elements.append(parts[0])
            c1.append([float(x) for x in parts[1:4]])
        else:
            c2.append([float(x) for x in parts[1:4]])
    return np.array(c1), np.array(c2), elements

def get_rigid_transform(p_start, p_end):
    """Calculates centroid and rotation (Kabsch) to move Start -> End."""
    cent_A = np.mean(p_start, axis=0)
    cent_B = np.mean(p_end, axis=0)
    P = p_start - cent_A
    Q = p_end - cent_B
    rot, _ = R.align_vectors(Q, P)
    return cent_A, cent_B, rot.as_rotvec(), P

def monte_carlo_swept_volume(frames, elements, n_samples):
    """Calculates the Union Volume of all frames using Monte Carlo."""
    print(f"Calculating volume using {n_samples} MC points...")
    # Flatten all frames to find the bounding box
    all_coords = np.vstack(frames)
    radii_list = np.array([RADII.get(e, 1.5) for e in elements])
    # We need max radius for the bounding box padding
    max_r = np.max(radii_list)
    min_b = np.min(all_coords, axis=0) - max_r - GRID_PADDING
    max_b = np.max(all_coords, axis=0) + max_r + GRID_PADDING
    box_vol = np.prod(max_b - min_b)
    rng = np.random.default_rng()
    pts = rng.uniform(min_b, max_b, size=(n_samples, 3))
    # Vectorized Check: Is point P inside ANY atom in ANY frame?
    mask = np.zeros(n_samples, dtype=bool)
    radii_sq = radii_list**2
    for frame in frames:
        # Check atoms in this frame
        for i, atom_pos in enumerate(frame):
            # Distance squared from this atom to all points
            d2 = np.sum((pts - atom_pos)**2, axis=1)
            mask |= (d2 < radii_sq[i])
    hit_ratio = np.sum(mask) / n_samples
    return hit_ratio * box_vol

# ==========================================
# 2. MAIN EXECUTION
# ==========================================
def main():
    print(f"Reading {INPUT_FILE}...")
    pos_A, pos_B, elements = read_xyz(INPUT_FILE)
    # --- A. Setup Rigid Bodies ---
    # Body 1 (Core)
    b1_start = pos_A[BODY1_INDICES]
    b1_end   = pos_B[BODY1_INDICES]
    c1_A, c1_B, r1_vec, shape1 = get_rigid_transform(b1_start, b1_end)
    # Body 2 (Ring)
    b2_start = pos_A[BODY2_INDICES]
    b2_end   = pos_B[BODY2_INDICES]
    c2_A, c2_B, r2_vec, shape2 = get_rigid_transform(b2_start, b2_end)
    # --- B. Generate Trajectory ---
    print(f"Generating {FRAMES} interpolated frames...")
```

```python
    all_frames = []
    # Forward Sweep
    for i in range(FRAMES):
        t = i / (FRAMES - 1)
        full_frame = np.zeros((ATOMS_PER_CATION, 3))
        # Interpolate Body 1
        curr_c1 = (1-t)*c1_A + t*c1_B
        curr_r1 = R.from_rotvec(r1_vec * t)
        full_frame[BODY1_INDICES] = curr_r1.apply(shape1) + curr_c1
        # Interpolate Body 2
        curr_c2 = (1-t)*c2_A + t*c2_B
        curr_r2 = R.from_rotvec(r2_vec * t)
        full_frame[BODY2_INDICES] = curr_r2.apply(shape2) + curr_c2
        all_frames.append(full_frame)
    # --- C. Volume Calculation ---
    swept_vol = monte_carlo_swept_volume(all_frames, elements, MC_SAMPLES)
    # Calculate Static Baseline (Volume of Frame 0)
    static_vol = monte_carlo_swept_volume([all_frames[0]], elements, MC_SAMPLES)
    print("\n" + "="*30)
    print(f"RESULTS (Rigid Body Interpolation)")
    print("="*30)
    print(f"Static Volume (Start): {static_vol:.2f} A^3")
    print(f"Swept Volume (Dynamic): {swept_vol:.2f} A^3")
    print(f"Expansion: {((swept_vol - static_vol)/static_vol)*100:.1f}%")
    print("="*30 + "\n")
    # --- D. Save Movie ---
    # Add reverse loop for smooth playback
    movie_frames = all_frames + all_frames[-2:0:-1]
    print(f"Saving movie to {OUTPUT_FILE}...")
    with open(OUTPUT_FILE, 'w') as f:
        for i, fr in enumerate(movie_frames):
            f.write(f"{ATOMS_PER_CATION}\nFrame {i}\n")
            for j, coords in enumerate(fr):
                f.write(f"{elements[j]} {coords[0]:.4f} {coords[1]:.4f} {coords[2]:.4f}\n")
    print("Done.")

if __name__ == "__main__": main()
```

Listing 2: Rigid-Body Swept Volume Script

3 Crystallographic Information Tables

Table S11: Crystallographic data and refinement details for compounds studied.

**Identification code**	**5NO$_2$Dime NTf$_2$**	**Dime NTf$_2$**	**MetThia NTf$_2$**	**BzMorph NTf$_2$**	**CHxDime NTf$_2$**
Empirical formula	C14H14F6N4O6S2	C14H15F6N3O4S2	C13H12F6N2O4S3	C14H18F6N2O5S2	C14H21N3O4F6S2
Formula weight	512.41	467.41	470.43	472.42	473.46
Temperature/K	150.00	101.00	150(2)	150.00	150.00
Crystal system	monoclinic	monoclinic	monoclinic	orthorhombic	triclinic
Space group	Cc	P21/n	P21/n	Pna21	P$\bar{1}$
a/Å	15.1546(5)	8.4955(5)	13.541(2)	18.4990(17)	6.6701(6)
b/Å	8.1469(2)	10.7132(6)	10.0589(17)	16.7277(17)	8.8785(6)
c/Å	16.2894(5)	20.7296(11)	13.634(3)	18.604(2)	17.3499(15)
α/°	90	90	90	90	94.204(3)
β/°	94.4020(10)	99.946(2)	102.797(7)	90	93.982(4)
γ/°	90	90	90	90	100.669(3)
Volume/Å^3	2005.21(10)	1858.33(18)	1810.9(6)	5757.0(10)	1003.37(14)
Z	4	4	4	12	2
ρ_{calc} g/cm^3	1.697	1.671	1.725	1.635	1.567
μ/mm^{-1}	0.362	0.373	0.493	0.364	0.346
F(000)	1040.0	952.0	952.0	2904.0	488.0
Crystal size/mm^3	0.632 × 0.263 × 0.255	0.226 × 0.147 × 0.037	0.264 × 0.238 × 0.207	0.22 × 0.14 × 0.13	0.26 × 0.24 × 0.14
Radiation	MoKα (λ = 0.71073)	MoKα (λ = 0.71073)	MoKα (λ = 0.71073)	MoKα (λ = 0.71073)	MoKα (λ = 0.71073)
2Θ range/°	5.682 to 61.188	4.294 to 56.664	6.128 to 61.21	4.404 to 66.368	5.432 to 66.454
Index ranges	$-21 \le h \le 19$, $-11 \le k \le 11$, $-23 \le l \le 23$	$-11 \le h \le 11$, $-14 \le k \le 14$, $-27 \le l \le 27$	$-19 \le h \le 19$, $-14 \le k \le 14$, $-19 \le l \le 19$	$-27 \le h \le 28$, $-25 \le k \le 25$, $-28 \le l \le 28$	$-10 \le h \le 10$, $-13 \le k \le 13$, $-26 \le l \le 26$
Reflections	20040	51814	46814	316245	24868
Independent refs	5845 [Rint = 0.0403, Rsigma = 0.0240]	4639 [Rint = 0.0797, Rsigma = 0.0308]	5555 [Rint = 0.0320, Rsigma = 0.0193]	21974 [Rint = 0.0517, Rsigma = 0.0217]	7494 [Rint = 0.0421, Rsigma = 0.0386]
Data/restraints/params	5845/2/291	4639/0/264	5555/13/311	21974/1609/1118	7494/1689/649
GOF on F^2	1.062	1.090	1.078	1.013	1.050
R indexes [I>=2σ (I)]	R1 = 0.0248, wR2 = 0.0647	R1 = 0.0400, wR2 = 0.0846	R1 = 0.0242, wR2 = 0.0624	R1 = 0.0394, wR2 = 0.0954	R1 = 0.0498, wR2 = 0.1298
R indexes [all data]	R1 = 0.0255, wR2 = 0.0654	R1 = 0.0515, wR2 = 0.0878	R1 = 0.0253, wR2 = 0.0632	R1 = 0.0554, wR2 = 0.1062	R1 = 0.0798, wR2 = 0.1482
Peak/hole / e Å^{-3}	0.25/-0.25	0.40/-0.35	0.32/-0.36	0.40/-0.48	0.39/-0.34
Flack parameter	-0.012(17)	-	-	0.46(7)	-

4 CrystalExplorer Interaction Energies

CrystalExplorer 25 was used to compute pairwise intermolecular interaction energies and energy-framework components at the CE-1p-B3LYP level of theory. CrystalExplorer reports the electrostatic, polarization, dispersion, and repulsion contributions that sum to a total interaction energy (E_{Tot}).

The tables below summarize the calculated interaction energies for the immediate cation coordination spheres in Dime[NTf_2] and 2MetThia[NTf_2].

4.1 Dime[NTf_2]

Table S12: CrystalExplorer interaction energies for Dime[NTf_2]. Energies are in kJ mol^{-1} and distances in Å.

**Label**	**Cnt**	**Dist**	**Description**	E_{Coul}	E_{Disp}	E_{Exch}	E_{Pol}	E_{Rep}	E_{Tot}
			Cation – Anion Interactions						
1	1	8.46	1A : 2A $1-x, -y, 1-z$	-211.9	-6.6	-10.3	-16.0	18.6	-226.8
2	1	7.34	1A : 2A $-1/2-x, 1/2+y, 3/2-z$	-169.9	-7.0	-4.1	-9.6	7.7	-182.5
3	1	8.40	1A : 2A $1/2+x, 3/2-y, -1/2+z$	-153.4	-9.7	-7.8	-9.2	13.8	-167.3
4	1	6.78	1A : 2A $-1/2-x, -1/2+y, 3/2-z$	-207.9	-11.7	-14.0	-20.2	25.4	-229.6
5	1	5.14	1A : 2A	-273.1	-22.5	-24.6	-34.4	45.0	-311.9
6	1	4.23	1A : 2A $1+x, y, z$	-274.5	-28.9	-27.4	-34.5	49.5	-319.1
7	1	8.98	1A : 2A $1-x, 1-y, 1-z$	-172.6	-4.6	-2.4	-7.7	4.4	-182.1
8	1	8.06	1A : 2A $1/2-x, -1/2+y, 3/2-z$	-151.7	-5.0	-3.1	-6.8	5.8	-160.5
9	1	8.54	1A : 2A $1/2-x, 1/2+y, 3/2-z$	-140.8	-3.5	-1.4	-4.9	2.8	-147.3
			Cation – Cation Interactions						
10	2	11.89	2A : 2A $1/2+x, 3/2-y, -1/2+z$	124.3	-1.6	-0.3	-4.2	0.5	119.5
11	2	10.53	2A : 2A $-1/2+x, 3/2-y, -1/2+z$	145.3	-4.4	-3.1	-7.8	5.1	135.6
12	1	8.50	2A : 2A $-1+x, y, z$	152.3	-1.7	-0.1	-5.3	0.1	146.5
13	2	5.58	2A : 2A $1/2-x, 1/2+y, 3/2-z$	170.8	-21.3	-22.0	-21.2	39.2	141.5

4.2 2MetThia[NTf_2]

Table S13: CrystalExplorer interaction energies for 2MetThia[NTf_2]. Energies are in kJ mol^{-1} and distances in Å.

**Label**	**Cnt**	**Dist**	**Description**	E_{Coul}	E_{Disp}	E_{Exch}	E_{Pol}	E_{Rep}	E_{Tot}
			Cation – Anion Interactions						
1	1	8.50	1A : 2A $x, y, 1+z$	-143.8	-4.0	-2.5	-5.3	4.7	-150.7
2	1	7.83	1A : 2A $1-x, 2-y, 1-z$	-159.1	-9.2	-6.0	-9.6	10.7	-173.5
3	1	6.48	1A : 2A	-213.1	-10.3	-10.1	-19.3	18.3	-234.3
4	1	6.36	1A : 2A $-1/2+x, 1/2-y, 1/2+z$	-223.7	-12.0	-16.1	-22.4	29.2	-246.5
5	1	5.52	1A : 2A $3/2-x, -1/2+y, -1/2-z$	-278.9	-21.7	-28.7	-35.6	52.9	-315.8
6	1	4.56	1A : 2A $1-x, 1-y, 1-z$	-271.2	-27.5	-33.1	-32.7	59.7	-310.9
7	1	10.06	1A : 2A $-1/2+x, -1/2-y, 1/2+z$	-160.0	-2.3	-0.4	-5.6	0.8	-166.4
8	1	8.03	1A : 2A $1/2+x, 1/2-y, 1/2+z$	-150.0	-3.4	-1.1	-5.9	2.1	-157.5
9	1	9.28	1A : 2A $x, -1+y, z$	-167.2	-4.1	-1.3	-6.9	2.7	-175.8

Continued on next page

Table S13 – continued from previous page

Label	Cnt	Dist	Description	E_{Coul}	E_{Disp}	E_{Exch}	E_{Pol}	E_{Rep}	E_{Tot}
			Cation – Cation Interactions						
10	2	10.06	2A : 2A $x, -1+y, z$	147.1	-4.8	-7.5	-7.7	13.0	139.0
11	2	7.36	2A : 2A $3/2-x, 1/2+y, 1/2-z$	172.7	-12.3	-14.0	-16.9	24.8	152.5
12	1	5.37	2A : 2A $1-x, 1-y, 1-z$	185.3	-21.9	-28.1	-23.3	50.0	156.1

5 Molecular Structure Data

The following table summarizes the Hirshfeld surface properties and torsion angles for the compounds studied.

Table S14: Molecular Structure Data. Hirshfeld Surface Volumes in Å^3, Hirshfeld Surface Areas in Å^2, and angles in degrees. Abbreviations: Glob. = Globularity, Asph. = Asphericity, Curv. = Curvedness.

**Compound**	**Anion**	**Vol.** (Å^3)	**Area** (Å^2)	**Glob.**	**Asph.**	d_{norm} **Range**	d_{norm} **Mean**	**Curv. Mean**	ϕ_1	ϕ_2
MimBr (A)	Br^-	228.13	230.13	0.785	0.179	-0.190 to 1.535	0.417	-0.954	116.36(13)	74.64(12)
MimBr (B)	Br^-	230.71	234.15	0.777	0.204	-0.217 to 1.371	0.428	-0.957	105.13(13)	57.74(13)
$5NO_2$Dime	NTf_2	296.23	280.49	0.766	0.138	-0.219 to 1.128	0.456	-0.945	162.63(17)	71.88(16)
Dime	NTf_2	256.64	250.53	0.780	0.170	-0.156 to 1.245	0.414	-0.954	95.30(18)	12.7(2)
Dime	I	248.79	241.00	0.794	0.174	-0.115 to 1.710	0.413	-0.981	118.68(7)	81.60(7)
CHxDime	NTf_2	274.28	254.56	0.802	0.181	-0.495 to 0.963	0.255	-0.942	107.6(4)	78.5(3)
									86.6(3)	41.2(3)
2MetThia	NTf_2	251.66	244.15	0.790	0.116	-0.336 to 1.204	0.350	-0.951	95.22(13)	13.54(14)
									94.7(5)	17.9(3)
Morph (1)	NTf_2	250.21	232.42	0.826	0.091	-0.310 to 1.159	0.378	-0.934	172.4(3)	90.3(3)
Morph (2)	NTf_2	270.14	241.23	0.838	0.083	-0.711 to 1.132	0.374	-0.968	168.6(8)	94.0(10)
									176.4(10)	76.1(15)
Morph (3)	NTf_2	249.20	231.07	0.829	0.096	-0.378 to 1.137	0.373	-0.928	169.5(3)	88.0(3)
Dime (Neutral)	–	132.85	143.53	0.877	0.060	–	–	–	–	–

6 Hirshfeld Surface Percentage Tables

The following tables provide the detailed breakdown of the Hirshfeld surface interactions. The diagonal header indicates the surface mapping: **Inside** elements are listed in the rows (vertical), and **Outside** elements are listed in the columns (horizontal).

6.1 MimBr

Table S15: Hirshfeld Surface Percentages for MimBr (Cation A)

Inside \ Outside	**H**	**C**	**N**	**Br**	**Total (%)**
**H**	60.1	9.5	1.5	11.6	82.7
**C**	10.7	2.6	0.9	0.4	14.6
**N**	1.7	0.9	0.0	0.1	2.6
**Br**	0.0	0.0	0.0	0.0	0.0
**Total (%)**	72.5	13.0	2.4	12.1	100.0

Table S16: Hirshfeld Surface Percentages for MimBr (Cation B)

Inside \ Outside	**H**	**C**	**N**	**Br**	**Total (%)**
**H**	58.0	9.9	1.5	13.4	82.8
**C**	11.4	2.6	0.8	0.1	14.9
**N**	1.5	0.8	0.0	0.0	2.3
**Br**	0.0	0.0	0.0	0.0	0.0
**Total (%)**	70.9	13.3	2.3	13.5	100.0

6.2 $5NO_2Dime[NTf_2]$

Table S17: Hirshfeld Surface Percentages for $5NO_2Dime[NTf_2]$

Inside \ Outside	**H**	**C**	**N**	**O**	**F**	**S**	**Total**
**H**	18.0	1.2	1.5	30.2	20.0	0.1	71.0
**C**	1.5	0.0	0.9	1.4	6.8	0.0	10.5
**N**	0.4	0.0	0.9	1.2	1.3	0.0	3.9
**O**	10.9	0.0	0.0	1.2	2.5	0.0	14.6
**F**	0.0	0.0	0.0	0.0	0.0	0.0	0.0
**S**	0.0	0.0	0.0	0.0	0.0	0.0	0.0
**Total (%)**	30.8	1.2	3.3	34.0	30.6	0.1	100.0

6.3 Dime[NTf_2]

Table S18: Hirshfeld Surface Percentages for Dime[NTf_2]

Inside \ Outside	**H**	**C**	**N**	**O**	**F**	**S**	**Total**
**H**	17.9	7.6	2.6	21.6	35.5	0.2	85.4
**C**	8.7	0.0	0.1	2.0	1.8	0.0	12.5
**N**	0.0	0.0	0.6	1.2	0.3	0.0	2.0
**O**	0.0	0.0	0.0	0.0	0.0	0.0	0.0
**F**	0.0	0.0	0.0	0.0	0.0	0.0	0.0
**S**	0.0	0.0	0.0	0.0	0.0	0.0	0.0
**Total (%)**	26.5	7.6	3.3	24.8	37.6	0.2	100.0

6.4 Dime[I]

Table S19: Hirshfeld Surface Percentages for Dime[I]

Inside \ Outside	H	C	N	I	Total (%)
**H**	64.8	6.8	1.5	10.5	83.6
**C**	6.6	6.2	1.0	0.0	13.8
**N**	1.5	1.1	0.0	0.0	2.6
**I**	0.0	0.0	0.0	0.0	0.0
**Total (%)**	72.9	14.0	2.5	10.5	100.0

6.5 2MetThia[NTf_2]

Table S20: Hirshfeld Surface Percentages for 2MetThia[NTf_2]

Inside \ Outside	H	C	N	O	F	S	Total
**H**	11.5	7.9	2.0	20.1	33.1	2.2	76.9
**C**	8.8	0.0	0.1	1.5	0.7	0.0	11.1
**N**	0.0	0.0	0.0	0.7	0.0	0.0	0.7
**O**	0.0	0.0	0.0	0.0	0.0	0.0	0.0
**F**	0.0	0.0	0.0	0.0	0.0	0.0	0.0
**S**	4.0	0.0	1.3	1.3	4.8	0.0	11.3
**Total (%)**	24.3	7.9	3.4	23.6	38.5	2.2	100.0

6.6 Morph[NTf_2]

Table S21: Hirshfeld Surface Percentages for Morph[NTf_2] (Cation A)

Inside \ Outside	H	C	N	O	F	S	Total
**H**	23.6	6.2	2.0	27.6	28.5	0.0	87.9
**C**	7.5	0.0	0.0	0.3	0.0	0.0	7.8
**N**	0.0	0.0	0.0	0.0	0.0	0.0	0.0
**O**	4.3	0.0	0.0	0.0	0.0	0.0	4.3
**F**	0.0	0.0	0.0	0.0	0.0	0.0	0.0
**S**	0.0	0.0	0.0	0.0	0.0	0.0	0.0
**Total (%)**	35.4	6.2	2.0	27.9	28.5	0.0	100.0

Table S22: Hirshfeld Surface Percentages for Morph[NTf_2] (Cation B)

Inside \ Outside	H	C	N	O	F	S	Total
**H**	23.8	7.0	3.0	25.5	29.6	0.0	88.9
**C**	6.7	0.0	0.0	0.1	0.0	0.0	6.9
**N**	0.0	0.0	0.0	0.0	0.0	0.0	0.0
**O**	4.2	0.0	0.0	0.0	0.0	0.0	4.2
**F**	0.0	0.0	0.0	0.0	0.0	0.0	0.0
**S**	0.0	0.0	0.0	0.0	0.0	0.0	0.0
**Total (%)**	34.8	7.0	3.0	25.6	29.6	0.0	100.0

Table S23: Hirshfeld Surface Percentages for Morph[NTf_2] (Cation C)

Inside \ Outside	H	C	N	O	F	S	Total
**H**	24.1	6.1	2.1	27.5	28.1	0.0	87.9
**C**	7.3	0.0	0.0	0.4	0.1	0.0	7.8
**N**	0.0	0.0	0.0	0.0	0.0	0.0	0.0
**O**	4.3	0.0	0.0	0.0	0.0	0.0	4.3
**F**	0.0	0.0	0.0	0.0	0.0	0.0	0.0
**S**	0.0	0.0	0.0	0.0	0.0	0.0	0.0
**Total (%)**	35.7	6.1	2.1	27.9	28.2	0.0	100.0

6.7 CHxDime[NTf_2]

Table S24: Hirshfeld Surface Percentages for CHxDime[NTf_2]

Inside \ Outside	H	C	N	O	F	S	Total
**H**	39.9	0.0	1.4	25.2	30.8	0.0	97.2
**C**	0.0	0.0	0.0	0.2	0.9	0.0	1.2
**N**	0.0	0.0	0.0	0.8	0.8	0.0	1.6
**O**	0.0	0.0	0.0	0.0	0.0	0.0	0.0
**F**	0.0	0.0	0.0	0.0	0.0	0.0	0.0
**S**	0.0	0.0	0.0	0.0	0.0	0.0	0.0
**Total (%)**	39.9	0.0	1.4	26.2	32.5	0.0	100.0

7 Atomic Displacement Parameters (PLATON)

Principal-axis ADPs (U_1, U_2, U_3) were extracted from PLATON. Equivalent isotropic displacement parameters (U_{eq}) and anisotropy ratios (U_3/U_1) are reported for comparison of local vibrational amplitudes and directional motion within the lattice.

7.1 Dime[NTf_2] Dataset

Table S25: PLATON-derived ADP principal components and derived metrics for [Dime][NTf_2]. Units for U-values are $Å^2$.

Atom	U_1	U_2	U_3	U_{eq}	U_3/U_1
S1	0.0115	0.0122	0.0145	0.0127	1.27
S2	0.0127	0.0142	0.0183	0.0151	1.43
F1	0.0131	0.0238	0.0436	0.0268	3.33
F2	0.0117	0.0289	0.0418	0.0275	3.57
F3	0.0123	0.0360	0.0427	0.0303	3.48
F4	0.0146	0.0256	0.0372	0.0258	2.55
F5	0.0135	0.0248	0.0438	0.0273	3.24
F6	0.0120	0.0411	0.0486	0.0339	4.04
O1	0.0142	0.0193	0.0248	0.0194	1.75
O2	0.0115	0.0233	0.0269	0.0206	2.34
O3	0.0135	0.0231	0.0329	0.0232	2.43
O4	0.0137	0.0236	0.0318	0.0230	2.33
N1	0.0121	0.0131	0.0158	0.0137	1.31
N3	0.0122	0.0154	0.0158	0.0144	1.29
N4	0.0111	0.0124	0.0178	0.0137	1.60
C2	0.0085	0.0152	0.0180	0.0139	2.11
C4	0.0113	0.0181	0.0210	0.0168	1.87
C5	0.0108	0.0187	0.0214	0.0170	1.97
C6	0.0113	0.0146	0.0230	0.0163	2.03
C7	0.0093	0.0132	0.0155	0.0127	1.66
C8	0.0130	0.0171	0.0203	0.0168	1.56
C9	0.0114	0.0202	0.0278	0.0198	2.44
C10	0.0113	0.0183	0.0293	0.0196	2.58
C11	0.0135	0.0217	0.0239	0.0197	1.77
C12	0.0127	0.0161	0.0174	0.0154	1.36
C13	0.0128	0.0254	0.0277	0.0219	2.17
C14	0.0106	0.0181	0.0333	0.0207	3.14
C15	0.0150	0.0180	0.0248	0.0193	1.65
C16	0.0150	0.0195	0.0220	0.0188	1.46

7.2 5-NO_2-Dime[NTf_2] Dataset

Table S26: PLATON-derived ADP principal components and derived metrics for [5-NO_2–Dime][NTf_2]. Units for U-values are $Å^2$.

Atom	U_1	U_2	U_3	U_{eq}	U_3/U_1
S1	0.0151	0.0170	0.0203	0.0174	1.35
S2	0.0149	0.0193	0.0219	0.0187	1.48
F1	0.0176	0.0367	0.0686	0.0410	3.89
F2	0.0177	0.0528	0.0717	0.0474	4.04
F3	0.0173	0.0353	0.0688	0.0405	3.99
F4	0.0179	0.0330	0.0704	0.0404	3.94
F5	0.0222	0.0308	0.0865	0.0465	3.90
F6	0.0168	0.0414	0.0531	0.0371	3.17
O1	0.0170	0.0365	0.0515	0.0350	3.02
O2	0.0234	0.0397	0.0464	0.0365	1.99
O3	0.0191	0.0283	0.0333	0.0269	1.74
O4	0.0150	0.0287	0.0321	0.0252	2.14
O5	0.0169	0.0263	0.0437	0.0290	2.59
O6	0.0162	0.0331	0.0345	0.0280	2.13
N1	0.0163	0.0179	0.0234	0.0192	1.44
N3	0.0138	0.0181	0.0297	0.0205	2.16
N4	0.0142	0.0187	0.0233	0.0187	1.65
N5	0.0182	0.0224	0.0391	0.0266	2.15
C2	0.0112	0.0203	0.0269	0.0195	2.39
C4	0.0177	0.0213	0.0256	0.0215	1.44
C5	0.0169	0.0183	0.0300	0.0217	1.78
C6	0.0147	0.0229	0.0294	0.0223	2.00
C7	0.0154	0.0192	0.0252	0.0199	1.64
C8	0.0192	0.0237	0.0396	0.0275	2.06
C9	0.0213	0.0331	0.0582	0.0375	2.73
C10	0.0237	0.0284	0.0572	0.0364	2.41
C11	0.0239	0.0300	0.0418	0.0319	1.75
C12	0.0200	0.0246	0.0321	0.0256	1.60
C13	0.0172	0.0269	0.0459	0.0300	2.66
C14	0.0198	0.0259	0.0316	0.0258	1.59
C15	0.0183	0.0231	0.0361	0.0258	1.97
C16	0.0171	0.0257	0.0332	0.0253	1.94

8 NMR Spectra

^{1}H, ^{13}C, and ^{19}F NMR analyses were performed on a Bruker 500 MHz NMR spectrometer at 295 K. Chemical shifts (δ) are reported in parts per million (ppm) and referenced to the residual ^{1}H signal of DMSO-d_6 as the solvent at room temperature.

8.1 Dime[NTf$_2$]

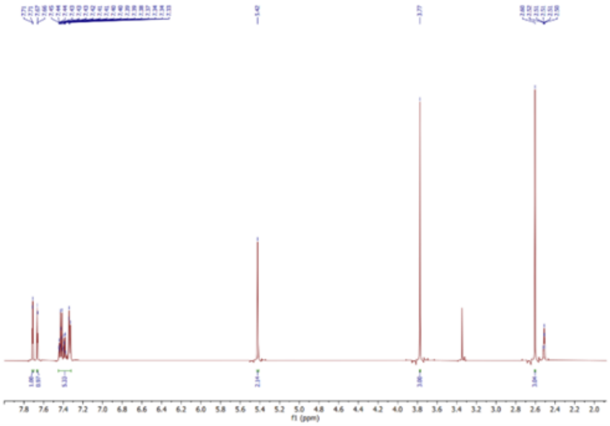

^{1}**H**

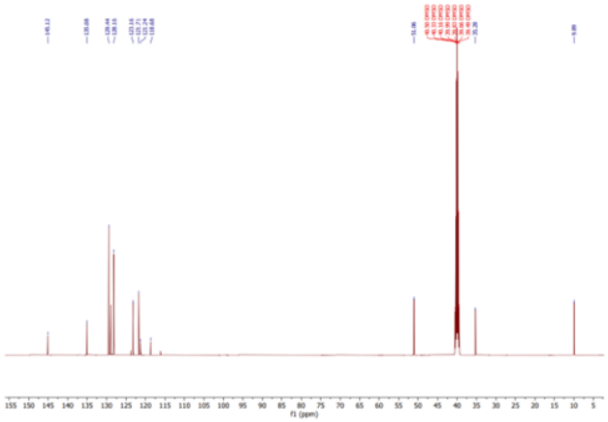

^{13}**C**

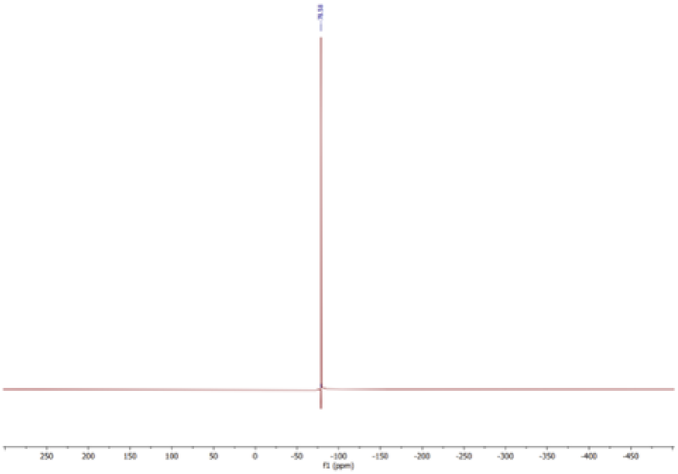

^{19}**F**

Figure S1: ^{1}H, ^{13}C, and ^{19}F NMR spectra of [Dime][NTf$_2$].

8.2 5-NO_2-Dime[NTf_2]

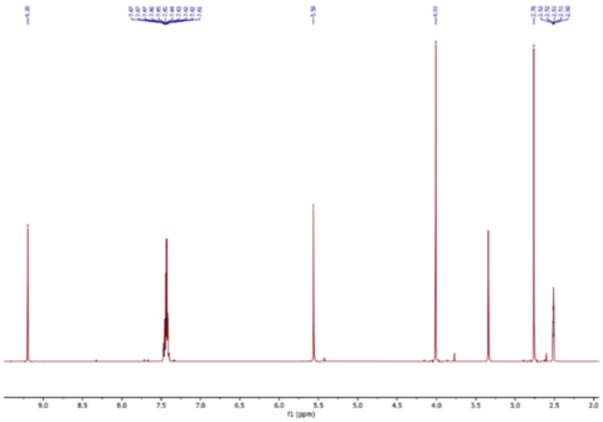

^{1}**H**

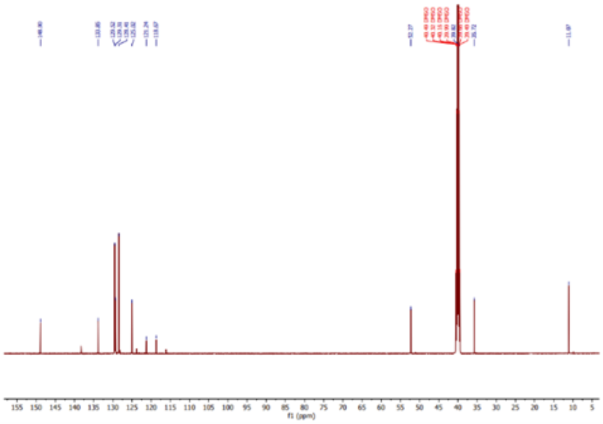

^{13}**C**

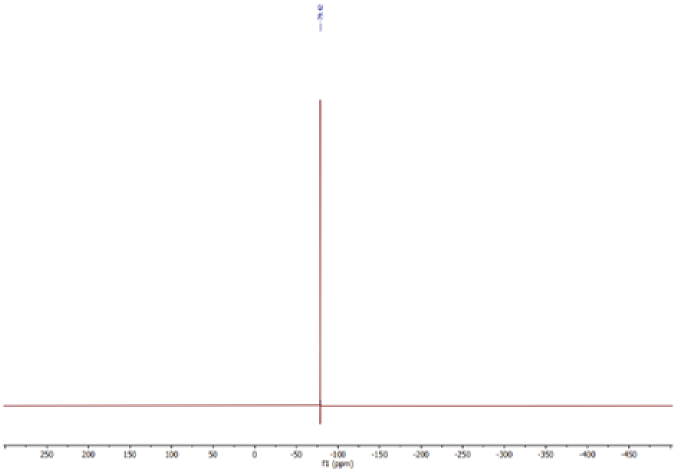

^{19}**F**

Figure S2: ^{1}H, ^{13}C, and ^{19}F NMR spectra of [5-NO_2−Dime][NTf_2].

8.3 2-MetThia[NTf_2]

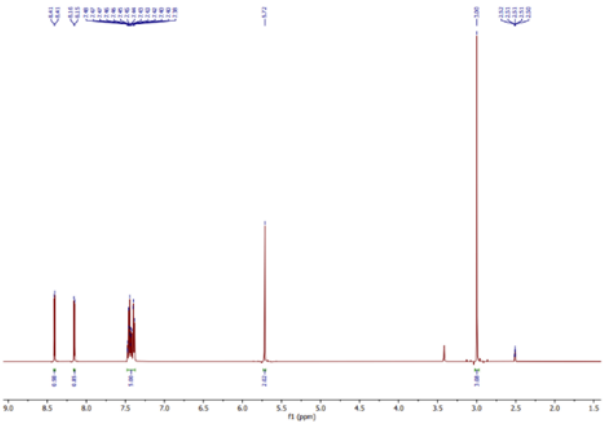

^{1}H

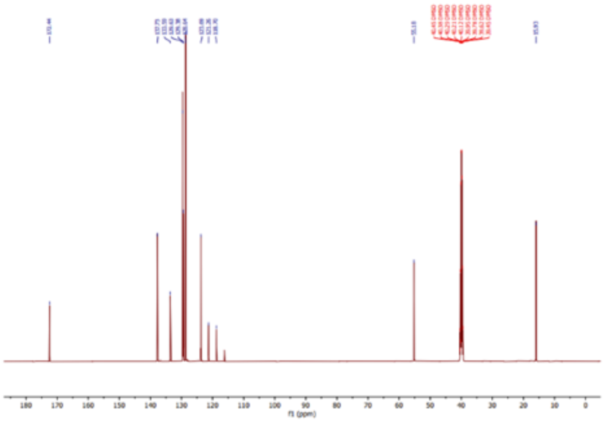

^{13}C

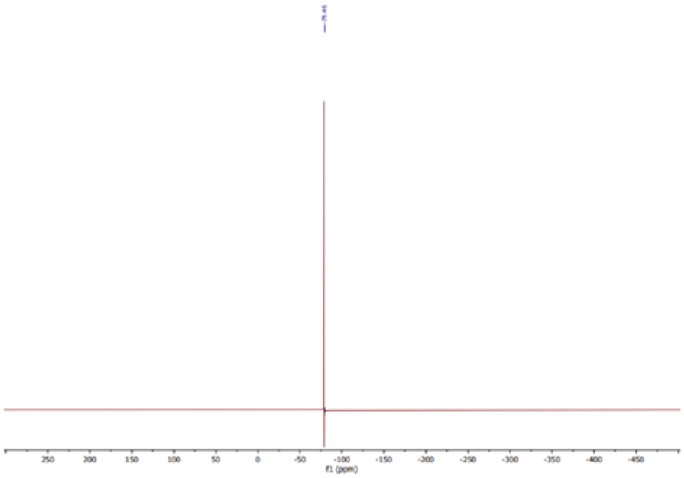

^{19}F

Figure S3: ^{1}H, ^{13}C, and ^{19}F NMR spectra of [2-MetThia][NTf_2].

8.4 Morph[NTf_2]

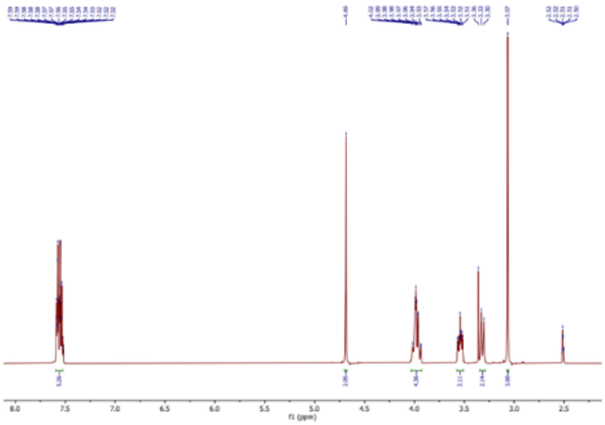

^{1}**H**

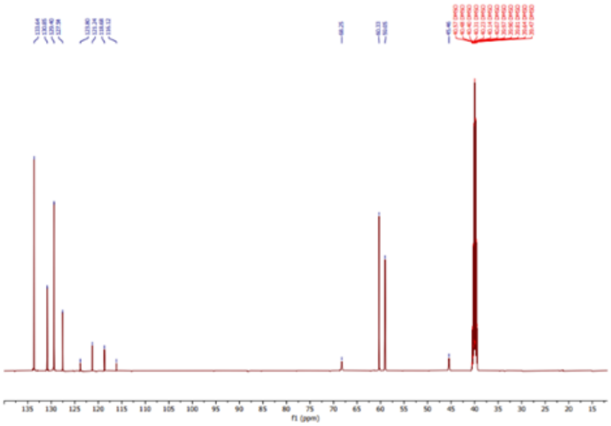

^{13}**C**

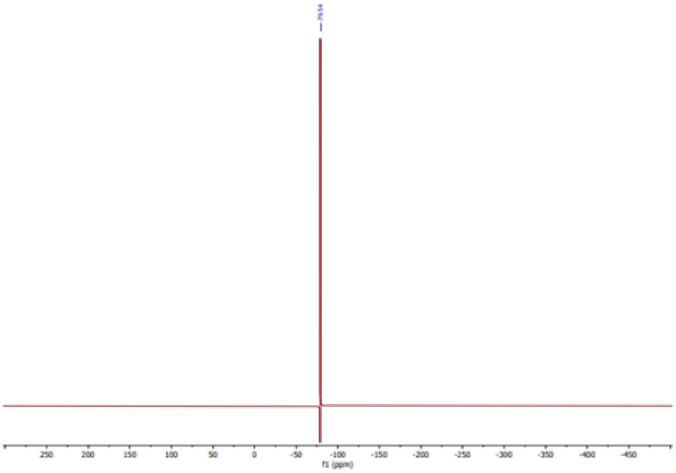

^{19}**F**

Figure S4: ^{1}H, ^{13}C, and ^{19}F NMR spectra of [Morph][NTf_2].

8.5 CHxDime[NTf_2]

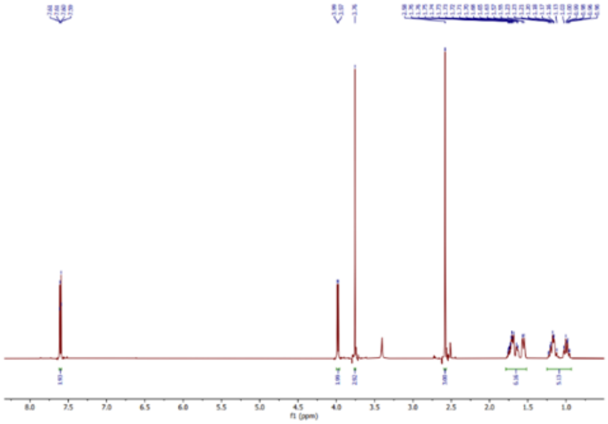

^{1}H

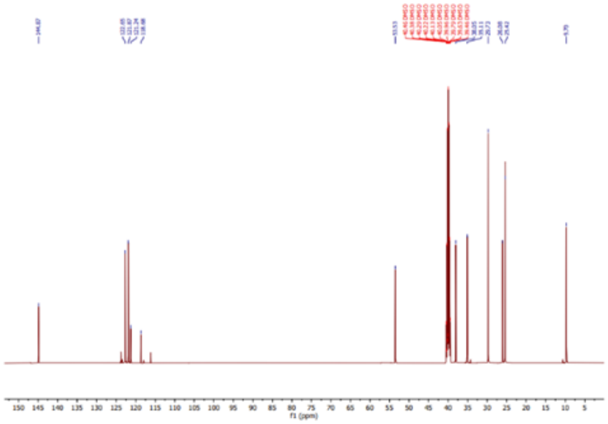

^{13}C

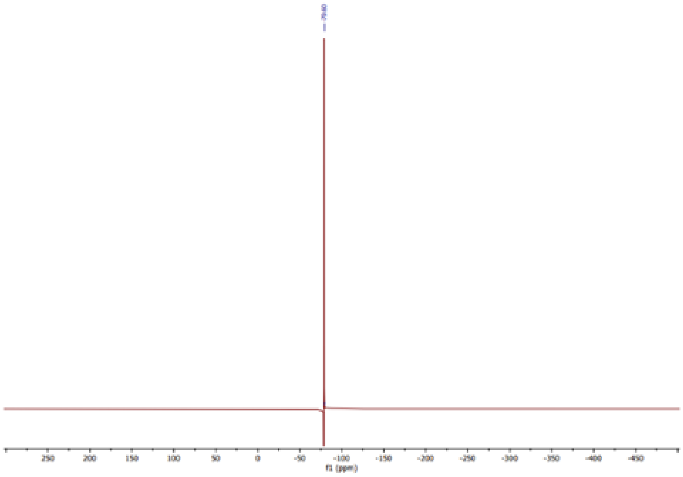

^{19}F

Figure S5: ^{1}H, ^{13}C, and ^{19}F NMR spectra of [CHxDime][NTf_2].
